# Balancing excitation and inhibition: The role of neural network dynamics in working memory gating

**DOI:** 10.1162/imag_a_00380

**Published:** 2024-12-02

**Authors:** Nadine Herzog, Elena Cesnaite, Paul Steinfath, Nikolai Kapralov, Sean J. Fallon, Vadim Nikulin, Arno Villringer, Lieneke K. Janssen, Annette Horstmann

**Affiliations:** Department of Neurology, Max Planck Institute for Human Cognitive & Brain Sciences, Leipzig, Germany; International Max Planck Research School on Neuroscience of Communication (IMPRS NeuroCom), Leipzig, Germany; Institute of Psychology, University of Münster, Münster, Germany; School of Psychology, University of Plymouth, Plymouth, United Kingdom; Institute of Psychology, Otto-von-Guericke University Magdeburg, Magdeburg, Germany; Department of Psychology, Faculty of Medicine, University of Helsinki, Helsinki, Finland; Collaborative Research Centre 1052, University of Leipzig, Leipzig, Germany

**Keywords:** working memory gating, network excitation–inhibition, long-range temporal correlations, 1/f slope, blood amino acid ratio, P300

## Abstract

In the complex landscape of daily life, we continuously balance between maintaining focus despite distractions and flexibly updating focus when needed—a cognitive process governed by a mechanism known as working memory gating. While much research has focused on the neural locus of this mechanism, less is known about the underlying neural dynamics. Here we probe the role of network excitation/inhibition (E/I) dynamics in working memory gating. Utilizing resting-state electroencephalography, we extract two markers of network E/I dynamics: resting-state long-range temporal correlations (LRTCs)—indicative of “critically” balanced E/I dynamics, and the slope of the power spectral density (PSD)—indicative of E/I ratio, and relate them to performance on a working memory gating task, specifically probing distractor-resistant maintenance and flexible updating. Based on previous studies linking stronger LRTCs to enhanced adaptive cognition, we initially expected to observe a similar relation. We find the opposite pattern, however: stronger LRTCs (indicating a more “critical” E/I balance) predicted poorer performance in maintenance-related working memory processes. This challenges the assumption that “near-critical” system dynamics are generally beneficial for cognitive function. Additionally, a flatter PSD slope (indicating a higher E/I ratio) was associated with better maintenance-related performance, particularly in individuals with higher levels of blood phenylalanine and tyrosine (indicating greater central dopamine availability). Notably, both network measures affected performance in all but the updating condition, suggesting a nuanced role of cortical E/I dynamics in overarching maintenance-related working memory processes, distinct from the gating mechanism as such. Our results highlight the complex interplay of network dynamics and neurochemical environments in cognitive function, suggesting implications for targeted interventions in cognitive disorders.

## 1 Introduction

In everyday life, we constantly need to flexibly adapt to an ever-changing complex environment. While in some situations one needs to be focused and resist distracting inputs, at other times it might be beneficial to let external inputs interfere with current mental representations. While reading these lines, for example, you need to concentrate and maintain current working memory (WM) contents despite possible distractions. Yet, you might also need to shift your attention if someone nearby mentions an urgent matter. A cognitive system enabling this adaptive coordination between distractor-resistant maintenance and updating of mental representations is WM gating (Chatham & Badre, 2015;Durstewitz et al., 2000). Prior studies, utilizing computational models and neurophysiological data, have successfully demonstrated the involvement of dopaminergic frontostriatal loops in these adaptive processes (Fallon & Cools, 2014;Fallon et al., 2017b;Landau et al., 2009). While the prefrontal cortex (PFC) plays a crucial role in sustaining representations in a distractor-resistant manner, the process of updating these is mediated by dopaminergic signaling in basal ganglia go/no-go pathways (Cools & D’Esposito, 2011;Landau et al., 2009). Rises in dopamine promote “go”-pathway activation via D1 receptors, facilitating updating. Lower dopamine levels activate the “no-go”-pathway via D2 receptors, supporting maintenance. Importantly, the effectiveness of phasic rises in dopamine to override tonic dopamine signals in the PFC has been shown to depend on initial baseline dopamine levels in the striatum (Cools & D’Esposito, 2011;Ranganath & Jacob, 2016;van Schouwenburg et al., 2010).

In addition to these dopaminergic mechanisms, the functional organization of neural networks in terms of excitatory and inhibitory (E/I) activity is also crucial for regulating working memory gating. As such, persistent maintenance of working memory contents in the PFC is governed by recurrent excitation within the network and inhibitory feedback regulation thereof (Durstewitz et al., 2000). Within frontostriatal loops, various excitatory and inhibitory connections balance adequate go-activation (triggering updating) or no-go activation (supporting maintenance; seeO’Reilly & Frank, 2006). Consequently, the precise interplay of excitatory and inhibitory signals is crucial for effective WM gating. However, the exact mechanisms by which these signals navigate distractor-resistant maintenance and selective updating remain elusive (see alsoDipoppa et al., 2016). One approach to examine excitation/inhibition (E/I) network dynamics is to analyze long-range temporal correlations (LRTCs) in ongoing oscillations from resting-state EEG. LRTCs describe the statistical dependency of signal fluctuations across different time scales, reflecting that the network may operate near the critical state—at the sweet spot between order and chaos (Hardstone et al., 2012;Linkenkaer‐Hansen et al., 2001). These critical‐state dynamics in neural oscillations have been linked to optimal information processing (Beggs & Plenz, 2004;Shew et al., 2011;Shew & Plenz, 2013), and deviations from this state have been associated with various cognitive dysfunctions, including depression (Linkenkaer-Hansen et al., 2005), schizophrenia (Moran et al., 2019;Nikulin et al., 2012), and epilepsy (Monto et al., 2007). LRTCs, particularly those derived from spontaneous oscillations during rest, exhibit high heritability (Linkenkaer-Hansen et al., 2007) and test–retest reliability (Nikulin & Brismar, 2004), making them a valuable marker for trait-like network characteristics. Importantly, these correlations emerge only when the excitatory and inhibitory activity across the underlying circuit is well balanced (Poil et al., 2012)—a relationship that has most comprehensively been characterized for the alpha frequency range (Fedele et al., 2016;Pfeffer et al., 2018;Poil et al., 2012). LRTCs from resting-state alpha oscillations hence pose an ideal candidate for inferring the intrinsic E/I network balance. Furthermore, previous research suggests that stronger resting-state alpha LRTCs are associated with better performance on tasks that probe higher-order brain functions such as attention (Irrmischer et al., 2017), sensorimotor processing (Samek et al., 2016), and adaptive decision making (Colosio et al., 2017), and, most importantly, phasic adaptive working memory (Mahjoory et al., 2019).

In addition to LRTCs, also the 1/f slope of the power spectral density (PSD) has been shown to be indicative of E/I network dynamics. Specifically, it reflects the ratio of excitatory and inhibitory activity across the circuit—the network’s E/I ratio (Gao et al., 2017). Animal studies have demonstrated that an increase in excitatory connections is associated with a flatter PSD slope, while an increase in inhibitory connections results in a steeper PSD slope (Gao et al., 2017; seeFig. 3A), determined by the relative abundance of glutamatergic and GABAergic connections, respectively. These findings have been extended to human EEG and MEG studies (Donoghue et al., 2020), show high reliability (Pathania et al., 2021), and were further supported by pharmacological interventions that manipulate the E/I ratio (Colombo et al., 2019;Lendner et al., 2020;Medel et al., 2023;Zsido et al., 2022).

In the present study, we utilize these two markers to investigate how E/I network dynamics relate to WM gating. We first examine their association with performance on a WM gating task, specifically probing ignoring versus updating of WM representations (seeFallon & Cools, 2014;Hartmann et al., 2023;N. Herzog et al., 2024a,2024b). Next, because WM gating heavily relies on dopaminergic signaling, we also explore the impact of blood amino acid ratio, that is the ratio of tyrosine and phenylalanine (precursors of dopamine) to other large neutral amino acids, on the observed relationships. This ratio is indicative of peripheral blood dopamine precursor availability and has been shown to relate to dopamine signaling in the striatum (Leyton et al., 2004;Montgomery et al., 2003; seeFig. 2A), and working memory gating performance (N. Herzog et al., 2024b). Lastly, we also probe whether effects of LRTCs or 1/f slope on WM gating may be explained by a more direct neural signature of WM gating, that is P300 amplitude at the moment of ignoring distractors versus updating. The P300 component is an event-related potential (ERP) that is commonly used as a neural marker for the allocation of attentional resources as well as updating of working memory representations (Gray et al., 2004;Polich, 2007).

Given previous reports, we hypothesized that (i) stronger resting-state alpha LRTCs will be associated with better overall WM performance, with more pronounced effects on updating, and that (ii) a steeper PSD slope—reflecting more inhibition—should foster better maintenance at the expense of updating. We further expect blood amino acid ratio to impact these relationships, and that both, LRTC and PSD slope, will influence P300 amplitude depending on the condition.

## 2 Materials and Methods

### 2.1 Participants

In total, 78 participants were recruited via the internal participant database of the Max Planck Institute for Human Cognitive and Brain Sciences (Leipzig, Germany) and via advertisements in public places and university facilities. Participants were specifically screened to exclude those with a history of clinical drug or alcohol abuse, neurological or psychiatric disorders, or first-degree relative history of neurological or psychiatric disorders. None of the participants had symptoms of depression at the time of the experiment, as assessed via a screening interview using the Structured Clinical Interview for DSM-IV (SCID;Wittchen et al., 1997). Five subjects had very noisy EEG data (see methods) and were excluded from the analysis. The final sample size thus was 73 participants (39 female; 34 male, 0 diverse). The mean age was 26.75 years (SD = 3.79, min = 20.10, max = 36.29). Mean estimated IQ was 108 (SD = 9.52, min = 74, max = 118) and mean BMI was 23.22 kg/m² (SD = 2.68, min = 18.92, max = 29.89). The study was conducted in compliance with the principles of the Declaration of Helsinki and was authorized by the Ethics Committee of the Medical Faculty at the University of Leipzig (172/19-ek). All participants provided written informed consent before participation and were financially compensated for their time.

### 2.2 Study design

This study was pre-registered athttps://osf.io/zdmkx(see transparency statement in the supplements for an explanation of deviations). After an initial screening via phone, participants were asked to come to the laboratory for two separate sessions (Supplementary Fig. S1). On the first day, they underwent the screening session where further inclusion and exclusion criteria were checked. After inclusion, serum blood samples were taken to extract information on amino acid profiles indicative of central blood amino acid ratio. Participants, therefore, came overnight-fasted. After the blood draw, participants filled in the Dietary Fat and free Sugar Questionnaire (DFS;Francis & Stevenson, 2013;Fromm & Horstmann, 2019), assessing their eating behavior and the BIS/BAS questionnaire (Behavioral inhibition and activation system scales;Carver & White, 1994;Strobel et al., 2006), assessing trait impulsivity. On the second test day, participants first did two neuropsychological tests: the digit span task (assessing baseline WM;Wechsler, 2008) and an IQ Test (“Wiener Matrizen Test-2”;Formann, Waldherr & Piswanger, 2011). After that, participants were prepared for the EEG. They first did a 10-min eyes-closed rest EEG recording. Participants were instructed to sit still, close their eyes, and stay awake. Participants then did a short training on two working memory tasks—only one of which is subject to this study (see pre-registration). Task order was counterbalanced. Upon completion of the training, participants were asked to explain the task in their own words. The experimenter had a checklist that entailed three major points participants had to mention (seeSupplementary Table S1). Participants could only proceed the actual experiment if they named all three points and had a training accuracy of >80%. All participants fulfilled these criteria on the first attempt. EEG activity was recorded during the working memory task. Upon completion of the tasks, participants were asked to indicate their level of tiredness and concentration felt during the task on a 10-point Likert scale.

### 2.3 Working memory task

The task used is the same as in previous studies conducted in our laboratory (Hartmann et al., 2023;N. Herzog et al., 2024a,2024b). It is a variation of a delayed match-to-sample task originally designed byFallon and Cools (2014), and comprises four conditions as shown inFigure 1A. In the ignore condition, participants first had to memorize a pair of patterns marked with a “T” for “target.” Subsequently, they are shown another pair of distraction patterns labeled with an “N” for “non-target,” which they must ignore. After that, participants are presented with a probe stimulus and had to determine whether it matches one of the first two target stimuli (“T”). In the update condition, participants again memorize a pair of “target” patterns (marked with a “T”). Then, they are presented with a new set of “target” patterns, which replace the previously presented stimuli as the target. Thus, at the probe phase after an update event, participants need to respond according to whether the probe image matches one of the second (updated) pair of stimuli. The two control conditions, which feature only one pair of stimuli, are matched to the temporal delay between encoding the to-be-matched targets and probe in the ignore and update conditions. The probe is presented for 2,000 msec. Each trial is separated by a fixed inter-trial interval of 2,000 msec. The task is separated into 4 blocks, each consisting of 32 trials spread evenly across the 4 conditions, totaling 128 trials. Feedback on average accuracy is presented after each block, serving as a small break for the participant. The stimuli are randomly computer-generated, monochromatic RGB “spirographs.” Outcome measure is accuracy. The total duration of the task is approximately 30 min.

**Fig. 1.**
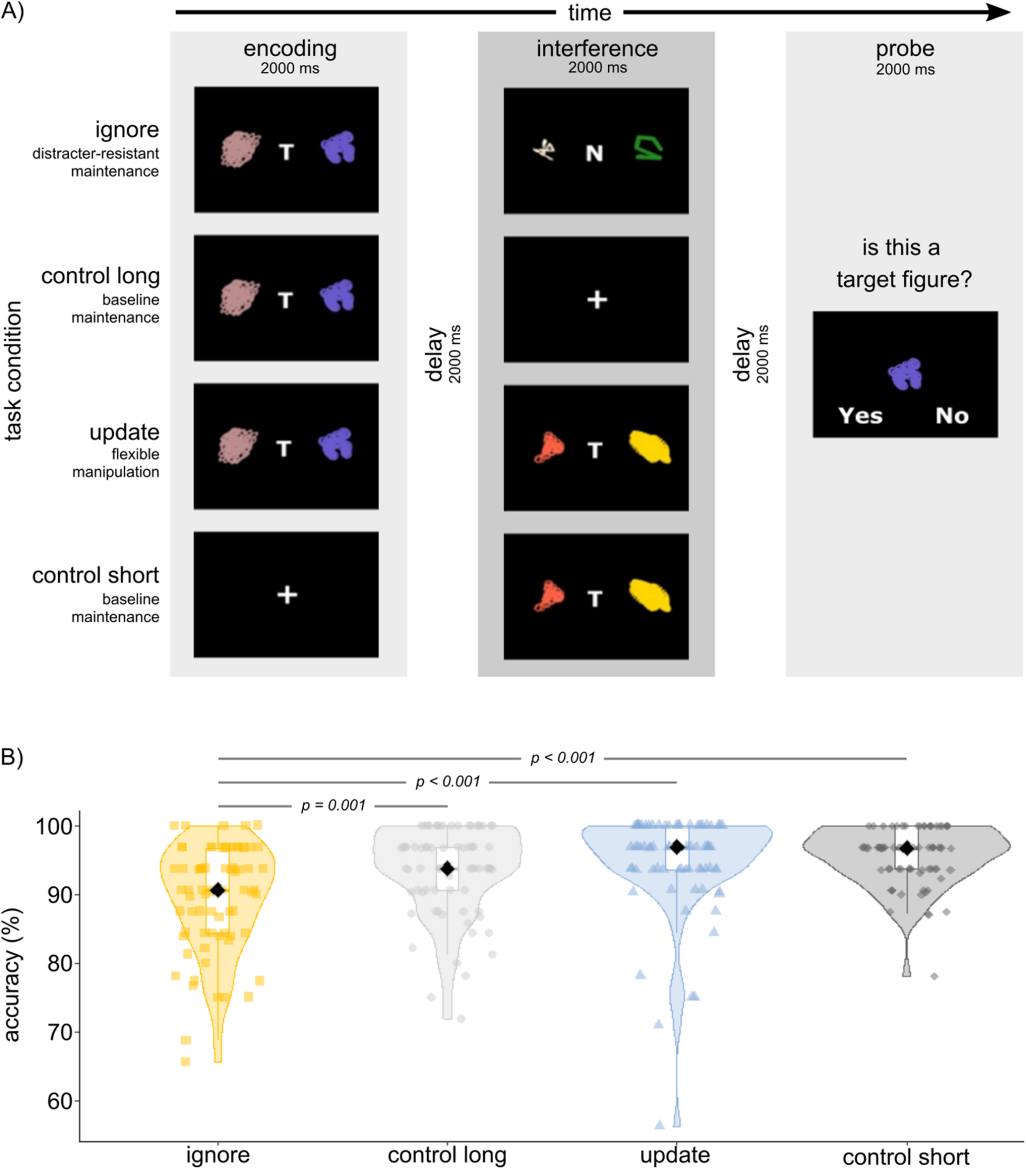
Task and general performance. (A) Schematic illustration of the task structure and experimental conditions. The task consists of three task phases. In the encoding phase, participants have to remember two target stimuli (signaled by the letter “T”), or are presented with a centered cross (short control trials). In the interference phase, participants either have to ignore two non-target stimuli (ignore trials; signaled by the letter “N”) or allow two new stimuli (again marked by a “T”) to replace the previously remembered target stimuli (update trials). No-interference trials (short and long control) do not require any manipulations in the interference phase. At the end of each trial, participants evaluate whether a presented figure was a target figure or not. Figure copied fromHartmann et al. (2023)with permission. (B) Accuracy per condition. Participants performed best in the short control condition (mean accuracy = 95.5%, SD = 4.2%), followed by update (mean = 94.5%, SD = 7.6%), control long (mean = 93.3%, SD = 6.4%), and ignore (mean = 89.5%, SD = 7.8%).

### 2.4 EEG recordings and pre‐processing

EEG data were recorded using 61-channel Ag/AgCl scalp electrodes (Brain Products GmbH, Gilching, Germany) in an EEG booth. The electrodes were mounted in an elastic cap (easyCAP, Herrsching, Germany) according to the international standard 10–20 extended localization system. The signal was amplified with a QuickAmp amplifier (Brain Products GmbH, Gilching, Germany). Additionally, two electrodes were placed above and beneath the right eye and recorded vertical (vEOG) and horizontal (hEOG) eye movements. One bipolar electrode attached to the right and left forearm recorded electrocardiogram (ECG). The electrodes were referenced to the common average reference with FCz being a ground electrode. The electrodes’ impedances were kept below 10 kΩ, the sampling rate was 1,000 Hz. EEG data were initially pre-processed using the MATLAB-based (MathWorks, Inc., Natick, MA, United States) EEGLAB toolbox (version 14.1.1b) and custom-written scripts. After downsampling the data to 500 Hz, it was band-pass filtered between 0.5 and 45 Hz (4th order Butterworth filter applied back and forth). In addition, a notch filter at 50 Hz was applied to remove any remaining power line artifacts. Flat channels and channels with correlation <0.8 with neighboring channels were removed using the*clean_artifacts()*function, and then interpolated. Bad epochs were detected using an adjusted version of the*trimOutlier()*function: We set different amplitude threshold levels for the noise detection at low-frequency (1–15 Hz) and high-frequency (15–45 Hz) ranges. For the low-frequency range, the individual noise threshold was defined as three SD above the mean amplitude of the filtered signal. We set a constant amplitude threshold of 40 μV for the high-frequency range given that noise in the high-frequency range is smaller in amplitude. Recordings for which the total bad segment length exceeded 60 s were inspected visually to confirm that the marked segments were indeed contaminated by noise. We excluded vEOG, hEOG, and ECG channels from the dataset and then applied independent component analysis (ICA, extended Infomax;Bell & Sejnowski, 1995). Components that reflect artifacts, such as muscle activity, non-biological noise, or eye-blinks, were marked and removed. Finally, we visually inspected the PSD of the multi-channel data of all subjects to determine whether data were sufficiently cleaned.

### 2.5 Resting-state EEG analysis

LRTCs were calculated using detrended fluctuation analysis (DFA;Hardstone et al., 2012). First, EEG signals were filtered into the alpha frequency range between 8 and 14 Hz and the amplitude envelope was extracted using the Hilbert transform. Then, the cumulative sum of the signal envelope was calculated. Finally, the root-mean-square fluctuation of the linearly detrended signal was calculated as a function of window size and plotted on double logarithmic axes. Time windows ranged between 2 and 25 s. Fitting a least-squares line eventually gives the DFA exponent, as reflected by the slope of the line. Notably, scaling exponents (α) in the range of 0.5–1.0 are generally considered indicative of persistent correlations indicating that the past of the signal affects its future, while a value of 0.5 indicates uncorrelated white noise (Cannon et al., 1997;Hardstone et al., 2012;Stam & de Bruin, 2004). The PSD of each channel’s data was calculated from the cleaned data using 4-second Hamming windows overlapping by 50% using Welch’s method. We used the Python (version 3.9) implementation of the FOOOF algorithm (Donoghue et al., 2020) on the PSD to estimate the slope of the 1/f decay for each channel separately: Here, broad-band power spectra between 3 and 40 Hz were modeled asP(f)∼1/fγ, where*γ*is the spectral slope. Additionally, we extract relative alpha power from the resting-state EEG to account for sleepiness. Relative alpha power was defined as the proportion of the alpha (8 to 13 Hz) wave activity compared with the total power of all frequencies.

### 2.6 Blood amino acid ratio

To determine the peripheral amino acid ratio, blood serum samples were collected from participants who had fasted overnight. These samples were analyzed at the Institut für Laboratoriumsmedizin, Klinische Chemie und Molekulare Diagnostik, University Clinic Leipzig. The blood amino acid ratio was calculated as:

$$aminoacidratio=\frac{Phenylalanine+Tyrosine}{Isoleucine+Lysine+Methionine+Tryptophan+Taurine+Valine}.$$

### 2.7 Statistical analyses

All behavioral analyses were done in RStudio v4.0.2 (R Core Team, 2015;RStudio Team, 2016). To assess whether intrinsic E/I network dynamics would relate to condition-dependent task accuracy, we ran linear mixed models using*lmer()*function from*lme4*package. The dependent variable in our models was accuracy. Before calculating average accuracy per condition, trials with a reaction time <200 ms were excluded, as those trials can be considered false alarms. Also, trials with a reaction time >2,000 ms were excluded, as those are considered misses. The predictor variables were the within-subject factor condition (ignore vs. update vs. control long vs. control short), and its interaction with the between-subject factor DFA exponents or PSD slope, respectively. To account for the within-subject nature of our data, we also included the random factor subject in all our models. Due to model convergence problems, we were not able to maximize the random structure of our model as recommended for linear mixed models (Barr, 2013). As we did not have any a priori hypothesis about the location of the effects, we ran separate models per electrode and did cluster-based permutation testing to correct for multiple comparisons (see below). We ran separate models for each network measure. Our models were:

(1) $$\;accuracy\;\sim \;condition\;*\;PSD_{electrode}+(1|subject)$$

(2) $$\;accuracy\;\sim \;condition\;*\;DFA_{electrode}+\left(1\;\;\;\text{|}\;subject\right).$$

Additionally, because WM gating depends on dopaminergic signaling, we further probed the influence of blood amino acid ratio on the relationship between intrinsic E/I network dynamics and WM gating in an exploratory manner. We, therefore, added blood amino acid ratio as factor of interest to the models:

(1a) $$\begin{array}{l} \;accuracy\;\sim \;condition\;*\;PSDelectrode\;*\;\\ \;\;\;\;\;\;\;amino\;acid\;ratio+\;(1|subject) \end{array}$$

(2a) $$\begin{array}{l} \;accuracy\;\sim \;condition\;*\;DFA_{electrode}\;*\;\;\;\\ \;\;\;\;\;\;\;\;amino\;acid\;ratio+\;(1|subject). \end{array}$$

To mitigate the issue of multiple comparisons in EEG analysis, we employed cluster-based permutation testing (Maris & Oostenveld, 2007). Specifically, as we ran one model per electrode, we identified electrodes in which DFA or PSD slope exhibited a significant condition-dependent association with task performance and grouped them into clusters. A group of electrodes was considered a cluster if it comprised at least two spatially adjacent electrodes that both demonstrated significant interaction effects at p < 0.05. The statistical significance of each identified cluster was then assessed by comparing its cumulative t-value against a null distribution of cumulative t-values generated by randomly resampling the data and recalculating the models 1,000 times. For every permutation, we determined and recorded the cumulative t-value of the largest spatially adjacent cluster. The cumulative t-value of our original cluster was then evaluated against this permutation-based null distribution. A cluster was deemed statistically significant if its cumulative t-value exceeded the 95th percentile of the permuted cumulative t-values, indicating that the observed clustering is unlikely to have arisen by chance under the null hypothesis.

Because DFA exponent, PSD slope, as well as task accuracy might partially be explainable by other confounding factors, we ran further control analyses. For this, we calculated a mean DFA and PSD slope exponent score across all electrodes within a cluster. These cluster averages were then used to re-run our models, incorporating possible confounders such as age, BMI, DFS, and BIS/BAS scores, task-related tiredness and concentration, sex, and IQ. Notably, since both the alpha DFA exponent and PSD slope may be affected by alpha power and SNR (e.g.,Linkenkaer-Hansen et al., 2007;Meisel et al., 2017;Pathania et al., 2021), we also included relative alpha power in the control model. By standardizing alpha power against total EEG power, we emphasize the effects of alpha-specific activity, rather than broadband difference in overall power, accounting for the individual contribution of alpha power. Additionally, this approach partially addresses variations in the data’s signal-to-noise ratio. Due to issues with model convergence, all continuous covariates were z-transformed.

### 2.8 Exploratory LRTC–PSD slope Interactions

**Fig. 2.**
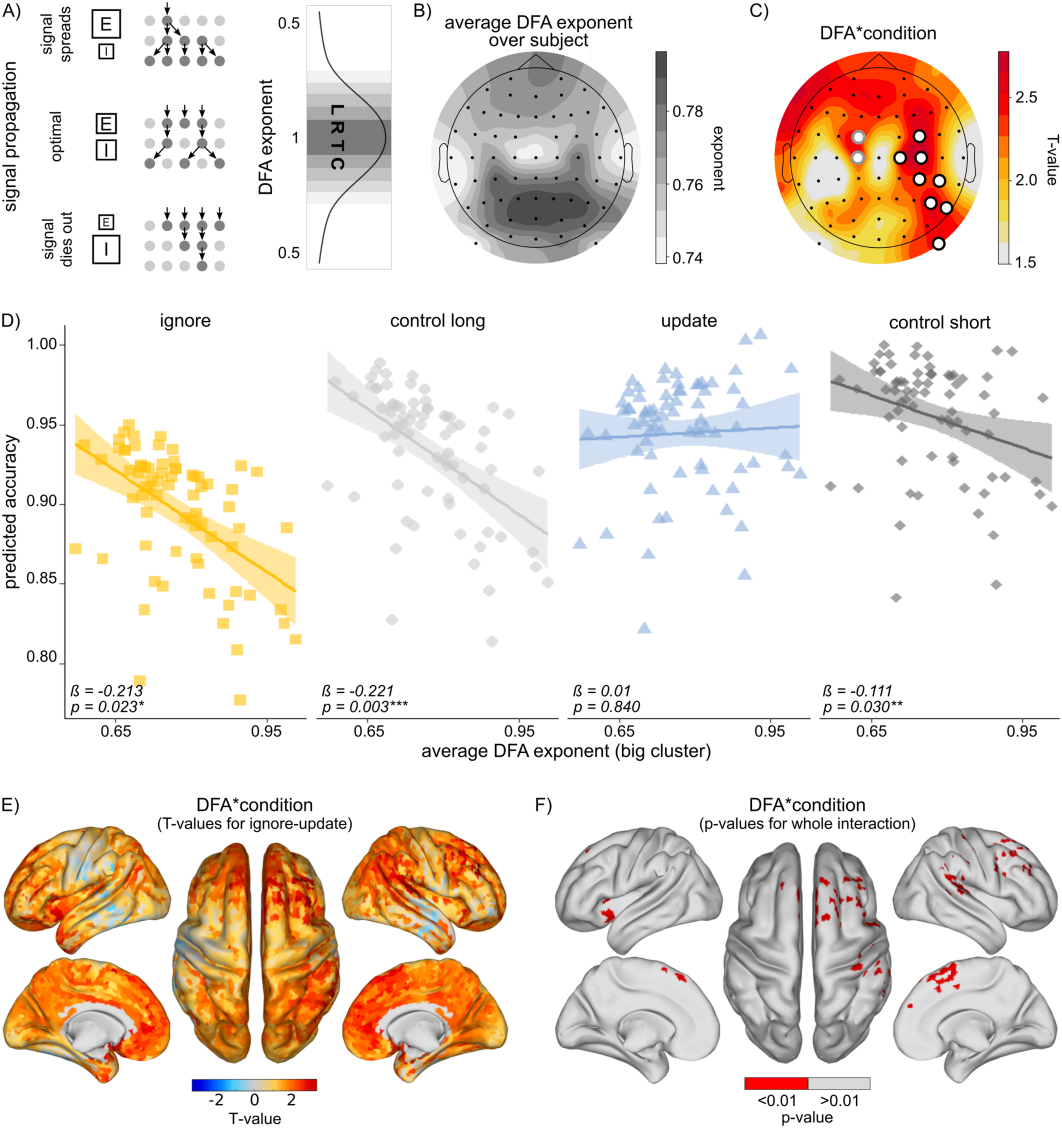
Effects of long-range temporal correlations (LRTCs) (A) Conceptual illustration of LRTCs and their relationship with signal propagation, excitation–inhibition (E/I) balance, and DFA exponents. Optimal signal propagation due to balanced E/I will be captured by a detrended fluctuation analysis (DFA) exponent close to 1, indicating LRTC emergence. Supra or suboptimal signal propagation due to imbalanced E/I will be captured by DFA exponent closer to 0.5. (B) Mean resting-state DFA exponents averaged across all subjects, illustrating the typical strength of LRTCs observed in our data. (C) Topographical map showing electrodes with significant interaction between DFA and condition. T-values for the contrast of interest (ignore–update) are plotted. Significant electrodes are marked with circles: white circles represent electrodes that survived cluster-based permutation testing. (D) Post hoc analysis results showing the effect of cluster-average DFA (from white-circled electrodes in panel C) on performance per condition. Average DFA relates negatively to task performance in ignore and control conditions, but not in the update condition. Data points reflect the fitted accuracy values per subject. Shaded areas indicate 95% confidence intervals for the regression lines. (E) Source analysis results displaying T-values for the contrast of interest (ignore–update), illustrating the spatial distribution of condition-dependent LRTC effects in the brain. (F) P-values for source–space results showing the interaction of DFA and condition per vertex. Significant vertices (p < 0.01, uncorrected) are highlighted in red. Significant areas include right frontal cortex, supramarginal gyrus, precentral gyrus, supplementary motor cortex, and parietal cortex, as well as left insular and orbitofrontal cortex.

Given that LRTC solely indicates whether the E/I balance is “optimal”, but not clearly discern whether the network leans more toward excitation or inhibition, we opted for an additional exploratory analysis. For this, we chose to look at how LRTCs and PSD slope would interactively predict condition-dependent task performance. This enabled an exploration of whether criticality in a more “excitable” sample (flatter PSD slope) would predict task performance differently than criticality in a more “inhibitory” sample (steeper PSD slope). For this, we build another average PSD slope value, based on those electrodes where LRTC significantly predicted condition-dependent performance (Fig. 2C, white circled electrodes). The final model included the dependent variable accuracy and the between-subject factors average DFA and average PSD slope, and the within-subject factor condition. For this analysis, we chose to look at our main conditions of interest only (ignore vs. update). The model was:

(3) $$\;accuracy\;\sim \;DFA_{avg}\text{​}*PSD_{avg}\text{​}*condition+\left(1\;\;\text{|}\;\;subject\right).$$

### 2.9 Source-level analysis

In order to gain a more detailed understanding of the origin of our effects, we repeated our sensor-level analysis (reported above; models 1, 2, 1a, and 2a) at the source level. We performed the analysis in Matlab R2022b, using the lead field matrix of the New York head model (Huang et al., 2016). A total of 4,502 sources with fixed orientations perpendicular to the cortical surface were considered. To match the preprocessing of the EEG data, a common average reference transform was applied to the lead field matrix (seeKapralov et al., 2023). The time series of activity for all sources were reconstructed using the eLORETA algorithm (exact low-resolution brain electromagnetic tomography), as implemented in the M/EEG Toolbox of Hamburg (METH, Guido Nolte; RRID: SCR_016104) for inverse modeling. We set the regularization parameter of eLORETA to 0.05 and used an identity matrix as the noise covariance matrix. The source-projected data were then filtered and transformed to compute the DFA and PSD slope exponent per vertex with exactly the same parameters used at the sensor level (Section 2.5). Subsequently, we used these values to perform vertex-wise analysis utilizing linear mixed models (lmer() in R v4.2.2). The models included the dependent variable average accuracy, and the within-subject predictor condition, and its interaction with the between-subject predictor source DFA exponent (model 4a) or source PSD slope and amino acid ratio (model 4b). For this analysis, we decided to refrain from correction methods, as it was a complementary analysis.

(4a) $$\;accuracy\;\sim \;condition\;*\;DFA_{vertex}+\;(1|subject)$$

(4b) $$\begin{array}{l} \;accuracy\;\sim \;condition\;*\;PSD_{vertex}\;*\;\\ \;\;\;\;\;\;\;\;amino\;acid\;ratio+\;\left(1\;\text{|}\;subject\right). \end{array}$$

### 2.10 Task-EEG analysis

Preprocessing of the task-EEG was done similar to resting-state EEG preprocessing. After that, task-EEG analysis was done using custom-written scripts in MNEmne Python (version 3.9). Continuous data were epoched based on task condition (ignore, update). Epochs extended from 500 ms pre-stimulus to 1,800 ms post-stimulus. Baseline correction was applied to each epoch using a pre-stimulus interval from -500 to -50 ms. After that, custom artifact rejection criteria were implemented to mark and reject remaining epochs with abnormal signal fluctuations: Epochs were marked if (i) more than 30% of the samples in a single channel exceeded an amplitude threshold of 15 µV, considering this as a sign of potentially artifactual activity, and if (ii) such activity was observed in more than two channels. After that, marked epochs were inspected visually and manually removed if they were indeed too noisy. On average, 4.93 epochs were removed per subject. Five subjects had too noisy task-EEG (>60% of epochs removed) and were, therefore, excluded from the analyses. By plotting the grand average ERP time series, we found that P300 amplitude (measured at Pz) peaked at around 320 msec. To capture this component, we thus extracted the average amplitude from 310 to 500 msec (Fig. 5A, gray area) per trial.

To check whether P300 amplitude would be a valid indicator of WM gating in our task, we ran trial-by-trial analysis using a logistic regression*glmer()*function from the lme4 package in R. We built the model using trial accuracy (binary; correct vs. incorrect) as outcome variable, and the within-subject factors P300 amplitude and condition (ignore vs. update) as interacting predictors. Trials with a reaction time <200 and >2,000 ms were excluded for this analysis. The model was

(5)   accuracy ~ P300 ∗ condition + (1 | subject).

To delve deeper into the influence of LRTCs or PSD slope on P300 amplitude, we performed further analysis utilizing trial-based linear mixed models (*lmer()*, lme4 package). Condition-dependent P300 amplitude was predicted by either cluster DFA (model 6a) or the combined effects of cluster PSD slope and amino acid ratio (model 6b).

(6a) $$\;P300\;\sim \;DFA_{cluster}\;*\;condition+(1\;\text{|}\;subject)$$

(6b) $$\begin{array}{l} \;P300\;\sim \;PSD_{cluster}\;*\;amino\;acid\;ratio\;*\;\\ \;\;\;\;\;\;\;\;condition\;+\;\left(1\;\text{|}\;subject\right). \end{array}$$

## 3 Results

### 3.1 General task performance

None of the participants performed below chance level (50% correct trials per condition, seeFig. 1B). Accuracy across conditions differed significantly (p_uncorrected_< 0.001). Participants performed best in the short control condition (mean accuracy = 95.5%, SD = 4.2%), followed by update (mean = 94.5%, SD = 7.6%), control long (mean = 93.3%, SD = 6.4%), and ignore (mean = 89.5%, SD = 7.8%).

### 3.2 Stronger LRTCs predict worse working memory performance

Next, to test whether WM gating performance would depend on intrinsic network dynamics in terms of E/I balance, we correlated resting-state DFA exponents to condition-dependent accuracy (see model 1 in Methods section). The grand average DFA exponent was 0.768 (SD = 0.014; min = 0.738; max = 0.796). Mean LRTC exponents averaged over subjects are presented inFigure 2B. We found that DFA exponents in 10 electrodes predicted condition-dependent task performance significantly (seeSupplementary Table S2, column DFA*condition). These electrodes formed two spatially adjacent clusters (Fig. 2C). Using cluster-based permutation testing, we then checked for significance of these clusters. The bigger, right frontoparietal cluster (C4, FC4, C2, CP4, CP6, P6, P8, PO10) was significant at p < 0.05. The smaller, frontocentral cluster (FC1, C1) was not significant (p > 0.05). SeeSupplementary Figure S2A. Control analyses indicated no influence of confounding variables such as age, sex, and BMI. (see Methods section) on this relationship. Notably, although relative alpha power had a significant main effect on overall task accuracy (p = 0.028), it did not diminish the primary effect of interest; the two-way interaction between mean DFA and condition remained significant. SeeSupplementary Table S3for the full model output.

To further investigate the observed DFA–condition interaction, we built a cluster-average DFA value and looked at its effect on accuracy per condition separately (Fig. 2D). These*post hoc*tests revealed that the initial interaction was likely due to there being a negative relationship between DFA exponents and accuracy in the ignore (β = -0.213, SE = 0.091, p_uncorrected_= 0.023) as well as in the two control conditions (β_control short_= -0.111, SE = 0.050, p_uncorrected_= 0.030; β_control long_= -0.221, SE = 0.074, p_uncorrected_= 0.003), but not in the update condition (β = 0.01; SE = 0.09; p_uncorrected_= 0.840).

In order to gain a more detailed understanding of the origin of DFA–condition interaction, we repeated the analysis done at the sensor level at the source level (see Methods section). Results reveal widespread effects (Fig. 2E). Sources significant at p < 0.01 (uncorrected) belong to the right frontal cortex, supramarginal gyrus, precentral gyrus, supplementary motor cortex, and parietal cortex, as well as left insular and orbitofrontal cortex (Fig. 2F). SeeSupplementary Table S4for a detailed list of all significant vertex clusters.*Post hoc*analyses looking at the effects of source DFA exponents (averaged over all vertices with p < 0.01) per condition show a pattern similar to that observed at the sensor level (Supplementary Fig. S3A).

Given the reliance of WM gating on dopaminergic signaling, we further probed the influence of blood amino acid ratio (as proxy for dopamine synthesis capacity) on the relationship observed above. Results show no significant three-way interaction in any of the electrodes (Supplementary Table S2, column DFA*condition*AA), suggesting that dopamine synthesis capacity does not moderate DFA effects on condition-dependent performance. Additional control analyses revealed that there was no direct correlation between blood amino acid ratio and resting-state DFA exponent (ß = 0.017; SE = 0.029; p = 0.541; seeSupplementary Fig. S5A).

### 3.3 Peripheral dopamine synthesis capacity moderates the effect of PSD slope on working memory gating

Next, we investigated how E/I ratio, that is resting-state PSD slope, relates to WM gating. Grand average PSD slope exponent was 1.779 (SD = 0.121; min = 1.604; max = 2.019). Mean PSD slope averaged over subjects is presented inFigure 3B. Correlating PSD exponents to condition-dependent accuracy (see model 2 in Methods section), we find no significant two-way interactions (Supplementary Table S2, column PSD*condition), indicating that PSD slope does not relate to condition-dependent task performance.

**Fig. 3.**
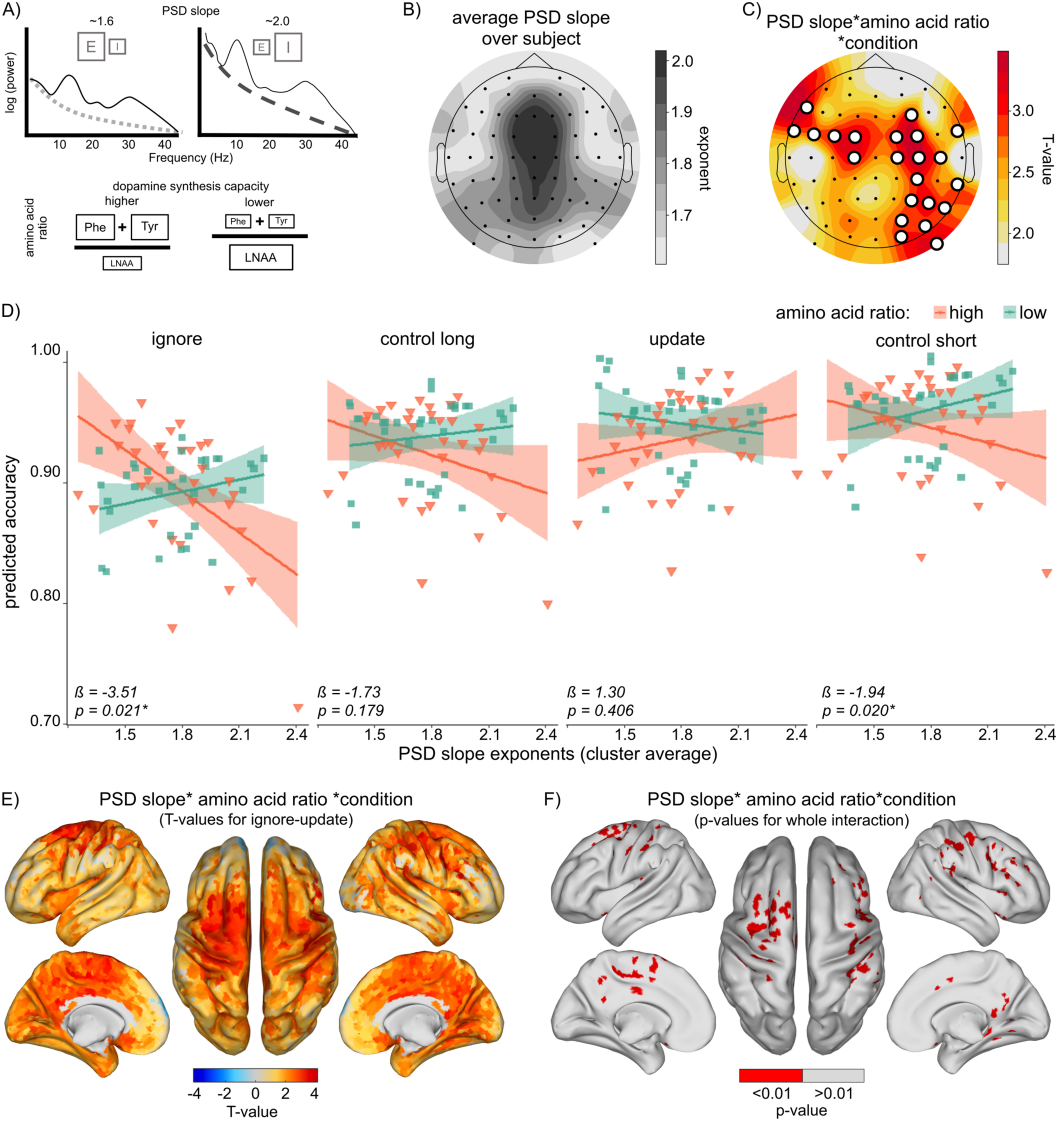
Power spectral density (PSD) Slope and Amino Acid Ratio Effects (A) Conceptual visualization of PSD slope and amino acid ratio. A flatter slope of the power spectrum indicates a higher E/I ratio (more excitation relative to inhibition), while a steeper slope indicates the opposite. Slope values shown are exemplary. Higher ratio of phenylalanine (Phe) and tyrosine (Tyr) relative to other large neutral amino acids (LNAA) indicates higher dopamine synthesis capacity. (B) Mean PSD slope averaged across all subjects, illustrating the typical slope observed in our data. (C) Topographical map showing significant electrodes for the interaction of PSD slope, amino acid ratio, and condition. T-values for the contrast of interest (ignore—update) are plotted. Significant electrodes are marked with circles. Both identified clusters survived cluster-based permutation testing. (D) Results from post hoc analyses examining the effect of cluster-average PSD slope–amino acid ratio interaction per condition. Significant interaction effects were found in the ignore and control short conditions, but not in the update condition. For visualization purposes, amino acid ratio was grouped into high and low categories based on a median split. PSD slope on the X-axis is an average of both clusters. Data points reflect the fitted accuracy values per subject. Shaded areas indicate 95% confidence intervals for the regression lines. (E) Source analysis results displaying T-values for the contrast of interest (ignore–update), illustrating the spatial distribution of condition-dependent PSD and amino acid ratio effects. (F) P-values for source–space results showing the interaction of PSD slope, amino acid ratio, and condition per vertex. Significant vertices (p < 0.01, uncorrected) are highlighted in red. Significant areas include right pre- and post-central gyri, frontal cortex, supramarginal gyrus, precuneous and parietal cortex, and cingulate gyrus, as well as left supplementary motor cortex, subcallosal cortex, precuneous cortex, cingulate gyrus, parahippocampal gyrus.

Interestingly, though, when adding amino acid ratio as a factor of interest, condition-dependent effects of PSD slope became apparent. We found significant three-way interactions in 22 electrodes (Supplementary Table S2, column PSD*condition*AA). These 22 electrodes could be divided into two spatially adjacent clusters (left frontocentral cluster: F7, FT7, FC5, FC3, FC1, C1; right frontoparietal cluster: F4, FC2, C4, P4, P8, O2, PO10, FC4, FT8, C2, C6, CP4, TP8, P6, PO4, PO8; seeFig. 3C). Permutation testing results showed that both clusters were significant at p < 0.05 (Supplementary Fig. S2B). Adding control variables such as age, sex, IQ, BMI, DFS, or BIS/BAS scores, or task-related tiredness and concentration to the models did not change these results. Importantly, while relative alpha power did show a significant main effect on overall task accuracy (p = 0.021), it did not take away from the main effect of interest, that is the three-way interaction of condition, PSD slope, and amino acid ratio stayed significant. SeeSupplementary Table S5for the full model output. Additional exploratory analyses revealed that there was no direct correlation between blood amino acid ratio and resting-state PSD slope exponent (ß = 0.007; SE = 0.028; p = 0.502; seeSupplementary Fig. S5B).

To further elaborate on the observed PSD—amino acid ratio—condition interaction, we built a cluster average PSD score per cluster and ran additional*post hoc*analyses, testing the interaction of cluster PSD and amino acid ratio per condition separately (Fig. 3D). These analyses show that the initial interaction was driven by a blood amino acid ratio—PSD slope interaction in ignore (β = -3.51, p_uncorrected_= 0.021) and control short (β = -1.94, p_uncorrected_= 0.020) conditions, but no such interaction in the update condition (β = 1.30, p_uncorrected_= 0.406). For the long control condition, the interaction was not significant (p_uncorrected_= 0.179), but the direction of effect aligned with ignore and control short conditions (β = -1.730). This effect was similar for both clusters.

Again, to get a better grasp of the origin of this effect, we repeated the analysis on the source level (see Methods section). Similarly to LRTCs, results show that the origin of the PSD slope, amino acid ratio, and condition interaction effect was widespread (Fig. 3E). Sources with p < 0.01 (uncorrected) were found in the right pre- and post-central gyri, frontal cortex, supramarginal gyrus, precuneous and parietal cortex, and cingulate gyrus, as well as left supplementary motor cortex, subcallosal cortex, and precuneous parahippocampal gyrus (cortex, cingulate gyrus;Fig. 3F). SeeSupplementary Table S4for a detailed list of all significant vertex clusters.*Post hoc*analyses of the interaction between PSD slope (averaged over all voxels with p < 0.01) and amino acid ratio per condition reveal a pattern similar to that observed at the sensor level (Supplementary Fig. S3B).

### 3.4 PSD slope shapes LRTC effects on task performance

**Fig. 4.**
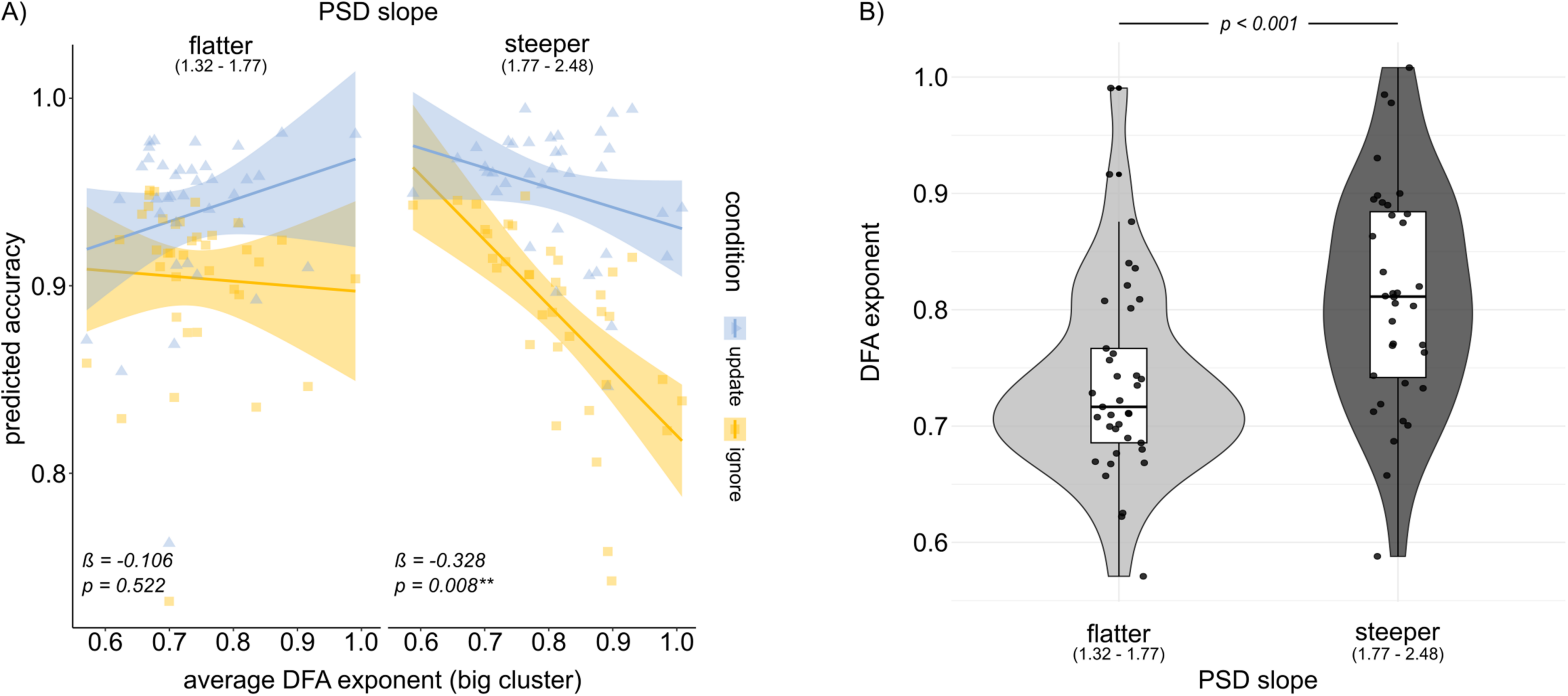
Interaction of cluster DFA and PSD slope. (A) In the subsample with a steeper PSD slope, that is more “inhibitory” network settings, DFA exponent correlates negatively with task performance, independent of condition (p = 0.008). In the subsample with a flatter PSD slope, that is more “excitatory” network settings, there was no significant correlation between DFA exponent and task performance (p = 0.522). Shaded areas indicate 95% confidence intervals for the regression lines. (B) DFA difference in PSD slope groups. Mean DFA was significantly lower in the flat-slope group than in the steep-slope group (p < 0.001).

Given that LRTC solely indicates whether the E/I balance is “optimal”, but does not clearly discern whether the network leans more toward excitation or inhibition, we opted for an additional exploratory analysis. For this, we chose to look at how LRTCs and PSD slope would interactively predict condition-dependent task performance. This way we could check whether criticality in a more “excitatory” sample (flatter PSD slope) would predict task performance differently than criticality in a more “inhibitory” sample (steeper PSD slope). Results reveal that indeed PSD slope and DFA predicted accuracy interactively (β = -1.096, SE = 0.4574, p = 0.016). This did not differ per condition, however (p_condition*DFA*PSD_= 0.406). Please refer toSupplementary Table S6for the full Anova Output. To dissect this DFA–PSD slope interaction further, we split the sample into steep versus flat PSD slope, based on median split, and looked at how DFA relates to accuracy in each of these subsamples. These analyses showed that DFA was negatively correlated with accuracy when PSD slope was steeper (β = -0.328; SE = 0.121, p = 0.008), while it did not correlate with accuracy when PSD slope was flatter (β = -0.106; SE = 0.164; p = 0.522;Fig. 4A). Additionally, in the sample with a flatter PSD slope (higher excitation), there was a trend significant interaction effect of condition and DFA (p = 0.071). The correlation between DFA and accuracy was numerically positive but insignificant in the update condition (β = 0.181; SE = 0.172, p = 0.299), while it was numerically negative and insignificant in the ignore condition (β = -0.106; SE = 0.156, p = 0.503). To further explore whether the interaction between DFA and PSD slope on accuracy might be associated with underlying differences in LRTCs across PSD groups, we checked whether DFA differs systematically between the two subgroups (Fig. 4B). Indeed, we found that in the subgroup with a steeper PSD slope, the mean DFA is significantly higher (mean = 0.812, SD = 0.095) than in the flat-slope PSD subgroup (mean = 0.737, SD = 0.0838, t(69.31) = -3.5784, p < 0.001), as shown inFigure 4B. To check whether this dependency leads to multicollinearity in our initial model (Model 3), we conducted a Variance Inflation Factor (VIF) analysis (see, e.g.,Thompson et al., 2017). The results showed a VIF of 1.10 for both PSD slope and DFA, indicating that there was no concerning collinearity among the predictors.

### 3.5 P300 amplitude reflects working memory gating, but is not affected by intrinsic E/I network dynamics

**Fig. 5.**
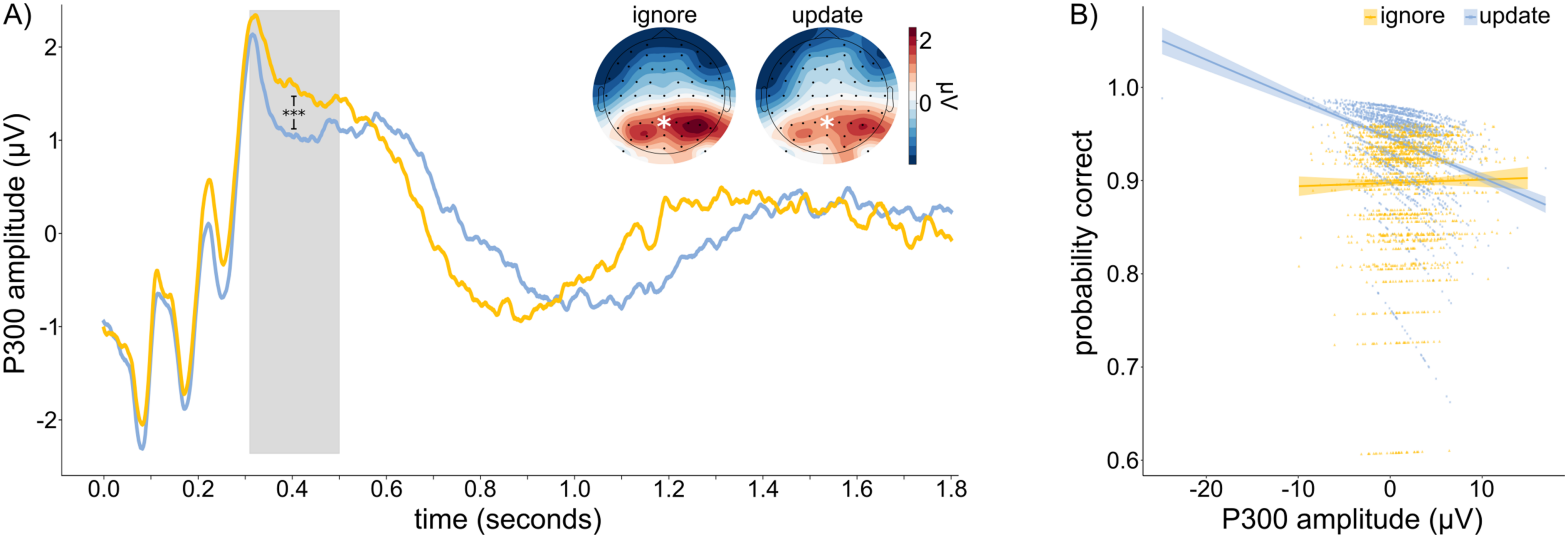
Effects of the P300 event-related potential on task performance. (A) P300 time series at PZ electrode per condition. The gray area marks the time window of interest from which we computed average P300 amplitude. Average amplitude in this time window was significantly higher for the ignore condition (mean = 1.651 μV, SD = 3.242 μV) than for the update condition (mean = 1.207 μV, SD = 3.356 μV, p < 0.001. (B) Relationship between P300 amplitude and condition-dependent task performance. After controlling for covariates, a significant interaction between condition and P300 amplitude was observed (p = 0.039). Post hoc analyses revealed a significant negative association between P300 amplitude and performance in the update condition (β = -0.071, SE = 0.031, p = 0.025), while there was no such association in the ignore condition (β = 0.003, SE = 0.024, p = 0.884).

To gain an insight into how LRTCs and 1/f slope influence behavioral outcomes, we examined their effects on brain activity during moments of ignoring or updating stimuli. For this, we extracted the P300 event-related potential (ERP) in response to the to-be-ignored or updated stimulus and first investigated whether its amplitude could predict condition-dependent task performance. Since higher P300 amplitude is generally associated with cognitive processes such as attention allocation (Kok, 2001;Polich, 2007) and working memory updating (Donchin & Coles, 1988;Verleger, 1988), we hypothesized that in our task, P300 amplitude would be higher in response to updating a stimulus compared with ignoring one. We observed the opposite pattern, however: average P300 amplitude was significantly higher in the ignore (mean = 1.651 μV, SD = 3.242 μV) than in the update condition (mean = 1.207 μV, SD = 3.356 μV, p < 0.001;Fig. 5A). Please refer toSupplementary Table S7for the full model output. Next, we tested whether this difference in amplitude could explain task performance. Results showed a trend-significant effect of P300 amplitude on accuracy, which varied by condition (condition*P300: β = -0.063, SE = 0.037, p = 0.086). After controlling for covariates (see Methods section), this effect became significant (β = -0.081, SE = 0.039, p = 0.039). SeeSupplementary Table S8for the full model output.*Post hoc*analyses show that P300 amplitude was negatively associated with update performance (β = -0.071, SE = 0.031, p = 0.025), while it was not associated with performance in ignore trials (β = 0.003, SE = 0.024, p = 0.884;Fig. 5B). As we conducted a trial-based analysis, we noticed that some P300 amplitudes were negative. To check whether these values indicated a shift in peak latency rather than true negative amplitudes, we split all trials into high- and low-amplitude groups, based on whether their amplitudes were above or below 0 μV, respectively. This follow-up analysis confirmed our suspicion (seeSupplementary Fig. S4), suggesting that our findings might be due to a combination of changes in amplitude and peak latency shifts. Finally, we investigated whether LRTCs or the PSD slope–amino acid ratio interaction (previously found to be relevant at the behavioral level) could predict condition-dependent P300 amplitude. Our analysis revealed that neither LRTCs nor the PSD slope–amino acid ratio interaction significantly predicted condition-dependent P300 amplitude (p_DFA * condition_= 0.146, p_PSD * amino acid ratio * condition_= 0.289). SeeSupplementary Tables S9 and S10for full model outputs, respectively.

## 4 Discussion

Balancing distracter-resistant maintenance and flexible updating of working memory representations is a key challenge for the brain and something that commonly goes awry in neuropsychiatric disorders (e.g.,Fallon et al., 2017a;Tanaka, 2006;Uitvlugt et al., 2016). Previous investigations have mainly focused on the neural locus of this gating process, demonstrating the involvement of prefrontal cortex and striatum (D’Ardenne et al., 2012;Fallon et al., 2017b;Nir-Cohen et al., 2020). The current study significantly advances these previous findings by exposing the E/I dynamics involved in working memory gating sub-processes. Specifically, we show that these processes can be predicted by two resting-state markers of E/I balance (LRTC) and E/I ratio (PSD slope), respectively.

### 4.1. Effects of cortical E/I dynamics are condition-specific

Interestingly, the effects of both LRTCs and PSD slope were particularly evident in the ignore, control long, and control short conditions, but not in the updating condition. This pattern is noteworthy as it highlights a shared characteristic among the affected conditions: despite interference in the ignore condition, all of them generally require the stable maintenance of WM representations over time. It seems, therefore, that network E/I dynamics primarily affect maintenance-related subprocesses of WM gating. This finding may be explained by the fact that maintenance processes are predominantly managed by the PFC (Frank et al., 2001;Hazy et al., 2006;O’Reilly & Frank, 2006). Since our study uses scalp EEG to capture LRTCs and PSD slope, these measures most likely reflect cortical dynamics, including those generated in the PFC, which is a cortical structure. In contrast, updating primarily relies on signaling of subcortical structures, specifically of the striatum (Hazy et al., 2006;O’Reilly & Frank, 2006;van Schouwenburg et al., 2010). The lack of significant effects of E/I dynamics on updating could, therefore, stem from methodological constraints, particularly the challenge of detecting striatal activation using scalp EEG. The condition-specific pattern observed in our data thus aligns well with literature pointing toward the engagement of different neural circuits underlying the two working memory gating processes. Our data provide further evidence for the dissociation of these processes at the level of cortical network dynamics.

Alternatively, the observed maintenance-specific pattern might suggest that cortical E/I dynamics affect overarching working memory processes rather than gating per se. Supporting this interpretation, we observed that neither LRTCs nor PSD slope influenced condition-dependent P300 amplitude—a more immediate marker of the gating process in our task.

### 4.2 Cortical E/I dynamics do not affect gating-related P300 amplitude

Given its condition-specific relationship to task performance, we conclude that P300 amplitude may serve as a valid marker for working memory gating in our task. Contrary to our expectations, however, the average P300 amplitude was higher in response to stimuli that should be ignored compared with those that required updating. Moreover, an increase in amplitude correlated with declining performance in update trials. At first glance, these findings seem to contradict established theories about P300, reflecting attention allocation and context updating (Donchin & Coles, 1988;Kok, 2001;Polich, 2007;Verleger, 1988). From a different perspective, however, P300 amplitude increases can also be interpreted as reflecting the number of cognitive resources utilized (Ghani et al., 2020). Our results hence suggest that ignoring irrelevant information may demand more cognitive resources, possibly because suppressing incoming information is more challenging. This interpretation gains support by our behavioral data, which indicate that participants generally performed worst in the ignore condition, implying a higher level of difficulty. In our paradigm, elevated P300 amplitude might, therefore, signify controlled inhibition, which would be detrimental in updating trials where participants should encode rather than inhibit stimuli. However, our data lack empirical support for this interpretation, preventing us from drawing definitive conclusions. Utilizing concurrent EEG-fMRI could help link P300 amplitude changes to specific brain regions involved in ignore and updating processes, potentially validating the proposed relationship between P300 and gating.

Nonetheless, the fact that neither LRTCs nor PSD slope affects this paradigm-specific marker of working memory gating suggests that cortical E/I network dynamics may influence task outcomes through mechanisms distinct from those reflected by P300-related gating. The precise pathways by which these cortical network properties modulate performance in our task thus remain an open and intriguing question, warranting further investigation.

### 4.3 Long-range temporal correlations relate negatively to working memory maintenance processes

Stronger LRTCs are frequently observed to correlate with improved cognitive performance (Nakao et al., 2019;Simola et al., 2017). Most important to our study,Mahjoory et al. (2019)found that stronger LRTCs were linked to faster and more accurate adaptive cognition in a working memory task—a finding we anticipated to extend to our study. Our results contradict these expectations, however, showing that LRTCs generally exhibited a negative relationship with WM task performance. Effect sizes for the significant negative relationships ranged from β = -0.11 to β = -0.22, indicating that even small increases in DFA exponents are associated with noticeable decreases in accuracy. Importantly, our control analysis, assessing the contribution of relative alpha power, suggests that the observed effects are unlikely to be confounded by individual alpha power differences, thereby highlighting the unique role of LRTCs.

The unexpected negative association between LRTCs and task performance might be explainable by theories suggesting that stronger LRTCs, which imply greater autocorrelation of the signal, go along with an increased signal “memory” (seeSimola et al., 2017). This concept proposes that higher LRTC scaling relations might lead to a prolonged influence of past neural states on current and future states. Providing empirical support for this concept,Smit et al. (2013)demonstrated that individuals with higher LRTC scaling showed a greater tendency to carry forward timing errors when attempting to maintain a steady rhythm in a fixed tapping task. Stronger temporal dependencies hence seem to lead to the carryover of past information. In the current study, participants are required to flexibly adapt to the demands of the task on a trial-by-trial basis, with no cues at the beginning of each trial to indicate whether it will be an ignore, update, or control trial. Only during the interference phase do participants become aware of the specific condition. In this adaptive task environment, stronger LRTC—reflecting greater signal “memory”—may hinder overall performance by causing interference between trials (also seeEuler et al., 2016). For example, features of an item seen in a previous trial may be carried over to the next trial, interfering with the mental representation of the current target item. In line with this reasoning, the negative effect of stronger LRTCs becomes particularly evident in maintenance-heavy conditions (ignore and controls) where discarding redundant information, such as presented distractors or (more generally) items seen in previous trials, is essential. Greater carryover of past information (i.e., greater signal memory) due to stronger LRTCs might thus disproportionately impact accuracy in these conditions. Updating, on the other hand, might not be affected by LRTCs, as it relies more heavily on rapid adaptation to changing information. The inherently adaptive nature of our task might prime an overly adaptive state, potentially rendering the influence of intrinsic signal memory (as represented by LRTC) less relevant for updating processes. Conversely, or in addition, the phasic increase of dopamine associated with updating may facilitate the selection of relevant information while suppressing outdated stimuli. This might counteract the potential interference caused by heightened signal memory, effectively reducing the negative impact of LRTCs on updating performance.

Interestingly, additional exploratory analyses examining the interaction between LRTCs and PSD slope suggest that the negative impact of strong LRTCs might be particularly detrimental to task accuracy in individuals with a steep PSD slope. Follow-up analyses revealed that this group generally exhibited higher DFA exponents, reflecting stronger LRTCs. The latter observation aligns with the network models proposed byPoil et al. (2012), which suggest that a lower E/I ratio generally results in temporal correlations closer to criticality (seeFig. 4AinPoil et al., 2012). In the context of our adaptive task, the combination of an inhibition-dominated network and stronger signal memory may lead to increased rigidity, which could explain the detrimental impact on task performance observed in this group. In contrast, participants with a flatter PSD slope exhibited lower baseline DFA exponents, indicating weaker LRTCs. The combination of weaker LRTCs (less signal memory) and a more excitable network (flatter PSD slope) may enable better adaptability by reducing the carryover of information from previous trials and enhancing responsiveness to new stimuli. In this more flexible neural state, the strength of LRTCs did not significantly affect task accuracy, suggesting that greater intrinsic network flexibility reduces the relevance of long-range dependencies.

Overall, it hence seems that a specific range of LRTC values can be either favorable or detrimental system dynamics, depending on the particular network predispositions and context (seeEuler et al., 2016;N. D. Herzog et al., 2021). Such interpretations should be taken with caution, however, as this was an exploratory analysis. Nevertheless, our results reinforce the notion that, despite potential state-dependent variations, resting-state LRTCs possess trait-like qualities, providing meaningful predictions of cognitive performance. Future research could build on these findings by manipulating the excitation–inhibition balance, potentially through pharmacological interventions, to investigate how changes in E/I balance affect the impact of LRTCs on cognition. Understanding these mechanisms in greater detail might inform therapeutic approaches to improve cognitive function in clinical populations.

### 4.4 PSD slope interacts with amino acid ratio to predict maintenance-related working memory processes

**Fig. 6.**
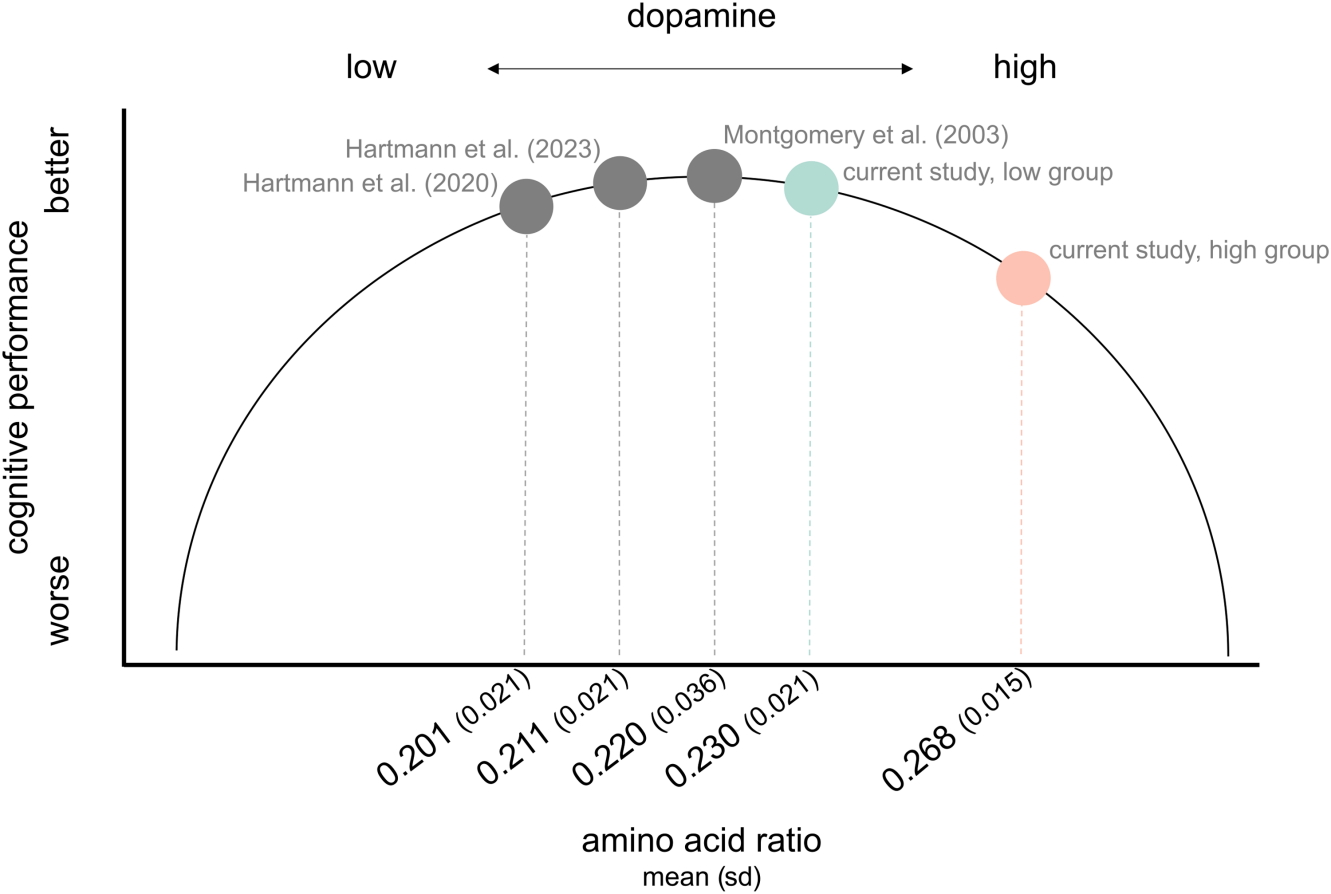
Conceptual illustration of the inverted U-shaped relationship between amino acid ratio (dopamine) and cognitive performance. Based on comparisons with mean ratios from other studies, the high amino acid ratio group (red) in our sample appears to fall on the right side of the dopamine-cognition parabola.

Contrary to our initial expectations, we found no significant direct association between PSD slope and condition-dependent task performance. However, when considering blood amino acid ratio, condition-specific effects of the PSD slope emerged. Specifically, for individuals with high amino acid ratios, there was a negative correlation between PSD slope and task performance in the three maintenance-related conditions (ignore, control short, and control long). There was no such relationship for low amino acid ratio individuals. The effect sizes for the significant interactions ranged from β = -1.73 to β = -3.51, indicating that even modest changes in amino acid ratio and PSD slope are associated with substantial decreases in task performance. Again, control analysis indicated no significant contribution of alpha power to these effects. Notably, the interaction effect was again absent in the update condition, reaffirming the maintenance specificity of cortical E/I dynamics. We speculate that our findings can be understood in light of the inverted U-shaped relationship between dopamine and PFC-dependent cognition (e.g.,Cools & D’Esposito, 2011): while intermediate dopamine levels foster good cognitive performance, extreme levels—both high and low—are associated with performance deterioration. A high amino acid ratio suggests an increased presence of dopamine precursors phenylalanine and tyrosine in the bloodstream (Moja et al., 1996), indicating heightened dopamine synthesis capacity and, consequently, elevated dopamine levels in the brain (Barrett & Leyton, 2004;Coull et al., 2012;Leyton et al., 2004;Montgomery et al., 2003). Comparing amino acid ratio ranges in our data with those from studies utilizing similar calculation methods (e.g., from our laboratory:Hartmann et al., 2020,^2023^; or externally:Montgomery et al., 2003), the high amino acid group in our sample appears to fall further right on the inverted U-shaped curve (Fig. 6). When dopamine levels are high, the PFC tends to operate in a D1-dominant state, which promotes heightened network stability (Durstewitz et al., 2000). This enhanced stability makes the neural circuits better equipped for maintaining working memory more resistant to distractions. However, this can come at a cost if inhibition becomes excessive, as indicated by a steeper PSD slope. The combination of a steep PSD slope and high amino acid ratios may, therefore, contribute to an “over-inhibition” state, resulting in an excessive suppression of neural activity. Given the adaptive nature of our task, as mentioned earlier, such a state could be detrimental to performance. At more middle range dopamine levels, however, variations in network E/I ratio might not have such a significant impact, as the system may be less prone to circuit stability.

Taken together, our findings hence indicate that the high amino acid ratio individuals in our sample are more sensitive to changes in network E/I ratio. This interpretation aligns with recent research indicating that excessive dopamine might disrupt the delicate balance between excitation and inhibition in the PFC, potentially via GABAergic signaling (seeDomenico & Mapelli, 2023).

### 4.5 Neural origin of effects

Given the specificity of our LRTCs and PSD slope effects to maintenance-related conditions, we anticipated that they might primarily be attributable to the dorsolateral prefrontal cortex (dlPFC). Indeed, source-level analysis partially confirmed the involvement of the dlPFC, with particularly pronounced effects in the right hemisphere. However, effects also extended to parietal areas, indicating the potential involvement of the frontoparietal attention network (FPN; e.g.,Scolari et al., 2015), a key system in cognitive control and working memory processes. Our findings are hence in line with previous studies suggesting the involvement of FPN in a variety of tasks that require phasic adaptive cognitive control (Eriksson et al., 2015;Mahjoory et al., 2019;T. P. Zanto & Gazzaley, 2013). Strikingly, the topography observed in our data largely resembles that reported byMahjoory et al. (2019), who also investigated the effects of resting-state LRTCs on working memory. Both, our study and that ofMahjoory et al. (2019), found a dominance of the right hemisphere, suggesting a consistent pattern of LRTC involvement in working memory processes across different experimental paradigms. This consistency strengthens the reliability of our findings and indicates a robust relationship between LRTCs in the frontoparietal network and working memory processes.

For PSD slope, the effects also spread to the left supplementary motor area (SMA), and some effects seemed to originate from the posterior cingulate cortex (PCC). The potential involvement of the PCC is particularly noteworthy, given its extensive interconnections with subcortical regions, including the basal ganglia (Vogt, 2019). This finding is intriguing as it aligns well with our observation of an interaction between PSD slope and amino acid ratio, that is dopamine synthesis capacity. As previously discussed, dopamine signaling in the basal ganglia is crucially involved in WM gating (e.g.,Cools & D’Esposito, 2011;Frank et al., 2001). The PCC’s involvement may thus represent a potential bridge between cortical dynamics (as measured by PSD slope) and subcortical dopaminergic processes influencing WM performance. The exploratory nature of our source-level analysis and the absence of multiple testing correction necessitate a conservative approach to drawing conclusions, however. Yet, the consistency between our sensor-level and source-level results lends credibility to these exploratory findings.

### 4.6 Broader implications and significance

Our findings significantly advance our understanding of the neural mechanisms underlying working memory gating processes. By demonstrating the role of resting-state cortical E/I dynamics in maintenance-related working memory processes, we provide new insights into how the brain manages specific aspects of information processing within the broader context of working memory gating. Our findings suggest that cortical E/I dynamics particularly influences the maintenance, rather than the updating component of gating. Moreover, our results challenge the broad assumption that stronger LRTCs would always be beneficial for cognitive functioning (also seeEuler et al., 2016; Fusca et al., 2023;Hardstone et al., 2012;Jäger et al., 2024), implying that they are either favorable or detrimental system dynamics, depending on the particular network predispositions and/or cognitive demands. Furthermore, our PSD slope findings underscore the importance of a delicate network E/I ratio for optimal cognition, complementing the established role of dopamine levels in working memory. These results have potential implications for understanding cognitive deficits in neuropsychiatric disorders characterized by working memory dysfunction. They suggest that alterations in cortical E/I dynamics could contribute to these deficits, opening new avenues for targeted manipulation studies and, consequently, interventions.

## Data and Code Availability

The data and code used to produce the presented results will be available athttps://github.com/O-BRAIN/WORMCRIupon publication.

## Author Contributions

N.H., L.K.J., E.C., and A.H. designed the study. N.H. and E.C. collected the data. N.H. analyzed the data. N.H. drafted the manuscript. Code review was done by P.S. Source-level analysis was done by N.K. E.C., P.S., N.K., S.J.F., V.N., A.V., L.K.J., and A.H. critically revised and approved the final manuscript.

## Funding

This research was supported by the FAZIT Foundation, the “Wiedereinstiegsstipendium” from the University of Leipzig, and funding from the Max Planck Society. The author gratefully acknowledges this financial support.

## Declaration of Competing Interest

No potential conflicts of interest relevant to this article were reported.

## Acknowledgements

The authors thank Sylvia Stasch and Lisa Okhof for helping with recruitment of participants and data collection. Furthermore, we thank the “Institut für Laboratoriumsmedizin, Klinische Chemie und Molekulare Diagnostik” at the University Clinic Leipzig for the analyses of our blood samples.

## Supplementary Materials

Supplementary material for this article is available with the online version here:https://doi.org/10.1162/imag_a_00380.

## Supplementary Material

Supplementary Material

## References

[b1] Barr , D. J. ( 2013 ). Random effects structure for testing interactions in linear mixed-effects models . Frontiers in Psychology , 4 , 54057 . 10.3389/fpsyg.2013.00328 PMC3672519 23761778

[b200] Barrett , S. P. , & Leyton , M. ( 2004 ). Acute phenylalanine/tyrosine depletion: a new method to study the role of catecholamines in psychiatric disorders . Primary psychiatry , 11 , 37 – 43 . http://accurateclinic.com/wp-content/uploads/2017/05/Acute-Phenylalanine-Tyrosine-Depletion-A-New-Method-to-Study-the-Role-of-Catecholamines-in-Psychiatric-Disorders-2004.pdf

[b2] Beggs , J. M. , & Plenz , D. ( 2004 ). Neuronal avalanches are diverse and precise activity patterns that are stable for many hours in cortical slice cultures . Journal of Neuroscience , 24 ( 22 ), 5216 – 5229 . 10.1523/jneurosci.0540-04.2004 15175392 PMC6729198

[b150] Bell , A. J. , & Sejnowski , T. J. ( 1995 ). An information-maximization approach to blind separation and blind deconvolution . Neural Computation , 7 ( 6 ), 1129 – 1159 . 10.1162/neco.1995.7.6.1129 7584893

[b201] Cannon , M. J. , Percival , D. B. , Caccia , D. C. , Raymond , G. M. , & Bassingthwaighte , J. B. ( 1997 ). Evaluating scaled windowed variance methods for estimating the Hurst coefficient of time series . Physica A: Statistical Mechanics and its Applications , 241 ( 3–4 ), 606 – 626 . 10.1016/S0378-4371(97)00252-5 PMC3204962 22049250

[b4] Carver , C. S. , & White , T. L. ( 1994 ). Behavioral inhibition, behavioral activation, and affective responses to impending reward and punishment: The BIS/BAS Scales . Journal of Personality and Social Psychology , 67 ( 2 ), 319 – 333 . 10.1037/0022-3514.67.2.319

[b6] Chatham , C. H. , & Badre , D. ( 2015 ). Multiple gates on working memory . Current Opinion in Behavioral Sciences , 1 , 23 – 31 . 10.1016/j.cobeha.2014.08.001 26719851 PMC4692183

[b7] Colombo , M. A. , Napolitani , M. , Boly , M. , Gosseries , O. , Casarotto , S. , Rosanova , M. , Brichant , J.-F. , Boveroux , P. , Rex , S. , Laureys , S. , Massimini , M. , Chieregato , A. , & Sarasso , S. ( 2019 ). The spectral exponent of the resting EEG indexes the presence of consciousness during unresponsiveness induced by propofol, xenon, and ketamine . NeuroImage , 189 , 631 – 644 . 10.1016/j.neuroimage.2019.01.024 30639334

[b8] Colosio , M. , Shestakova , A. , Nikulin , V. V. , Blagovechtchenski , E. , & Klucharev , V. ( 2017 ). Neural mechanisms of cognitive dissonance (revised): An EEG study . Journal of Neuroscience , 37 ( 20 ), 5074 – 5083 . 10.1523/jneurosci.3209-16.2017 28438968 PMC5444193

[b9] Cools , R. , & D’Esposito , M. ( 2011 ). Inverted-U–shaped dopamine actions on human working memory and cognitive control . Biological Psychiatry , 69 ( 12 ), e113 – e125 . 10.1016/j.biopsych.2011.03.028 21531388 PMC3111448

[b10] Coull , J. T. , Hwang , H. J. , Leyton , M. , & Dagher , A. ( 2012 ). Dopamine precursor depletion impairs timing in healthy volunteers by attenuating activity in putamen and supplementary motor area . Journal of Neuroscience , 32 ( 47 ), 16704 – 16715 . 10.1523/jneurosci.1258-12.2012 23175824 PMC6621775

[b11] D’Ardenne , K. , Eshel , N. , Luka , J. , Lenartowicz , A. , Nystrom , L. E. , & Cohen , J. D. ( 2012 ). Role of prefrontal cortex and the midbrain dopamine system in working memory updating . Proceedings of the National Academy of Sciences of the United States of America , 109 ( 49 ), 19900 – 19909 . 10.1073/pnas.1116727109 23086162 PMC3523834

[b13] Dipoppa , M. , Szwed , M. , & Gutkin , B. S. ( 2016 ). Controlling working memory operations by selective gating: The roles of oscillations and synchrony . Advances in Cognitive Psychology , 12 ( 4 ), 209 . 10.5709/acp-0199-x 28154616 PMC5280056

[b15] Di Domenico, D. , & Mapelli , L. ( 2023 ). Dopaminergic modulation of prefrontal cortex inhibition . Biomedicines , 11 ( 5 ), 1276 . 10.3390/biomedicines11051276 37238947 PMC10215621

[b16] Donchin , E. , & Coles , M. G. H. ( 1988 ). Is the P300 component a manifestation of context updating? Behavioral and Brain Sciences , 11 ( 3 ), 357 – 374 . https://www.cambridge.org/core/journals/behavioral-and-brain-sciences/article/abs/is-the-p300-component-a-manifestation-of-context-updating/F8EE26F6C82EA63B167C6E2A497C63D6

[b17] Donoghue , T. , Haller , M. , Peterson , E. J. , Varma , P. , Sebastian , P. , Gao , R. , Noto , T. , Lara , A. H. , Wallis , J. D. , Knight , R. T. , Shestyuk , A. , & Voytek , B. ( 2020 ). Parameterizing neural power spectra into periodic and aperiodic components . Nature Neuroscience , 23 , 1655 – 1665 . 10.1038/s41593-020-00744-x 33230329 PMC8106550

[b18] Durstewitz , D. , Seamans , J. K. , & Sejnowski , T. J. ( 2000 ). Dopamine-mediated stabilization of delay-period activity in a network model of prefrontal cortex . Journal of Neurophysiology , 83 ( 3 ), 1733 – 1750 . 10.1152/jn.2000.83.3.1733 10712493

[b19] Eriksson , J. , Vogel , E. K. , Lansner , A. , Bergström , F. , & Nyberg , L. ( 2015 ). Neurocognitive architecture of working memory . Neuron , 88 ( 1 ), 33 – 46 . 10.1016/j.neuron.2015.09.020 26447571 PMC4605545

[b20] Euler , M. J. , Wiltshire , T. J. , Niermeyer , M. A. , & Butner , J. E. ( 2016 ). Working memory performance inversely predicts spontaneous delta and theta-band scaling relations . Brain Research , 1637 , 22 – 33 . 10.1016/j.brainres.2016.02.008 26872594

[b21] Fallon , S. J. , & Cools , R. ( 2014 ). Reward acts on the pFC to enhance distractor resistance of working memory representations . Journal of Cognitive Neuroscience , 26 ( 12 ), 2812 – 2826 . 10.1162/jocn_a_00676 24893740

[b22] Fallon , S. J. , Mattiesing , R. M. , Muhammed , K. , Manohar , S. , & Husain , M. ( 2017a ). Fractionating the neurocognitive mechanisms underlying working memory: Independent effects of dopamine and Parkinson’s disease . Cerebral Cortex , 27 ( 12 ), 5727 – 5738 . 10.1093/cercor/bhx242 29040416 PMC5939219

[b23] Fallon , S. J. , van der Schaaf , M. E. , ter Huurne , N. , & Cools , R. ( 2017b ). The neurocognitive cost of enhancing cognition with methylphenidate: Improved distractor resistance but impaired updating . Journal of Cognitive Neuroscience , 29 ( 4 ), 652 – 663 . 10.1162/jocn_a_01065 27779907

[b24] Fedele , T. , Blagovechtchenski , E. , Nazarova , M. , Iscan , Z. , Moiseeva , V. , & Nikulin , V. V. ( 2016 ). Long-range temporal correlations in the amplitude of alpha oscillations predict and reflect strength of intracortical facilitation: Combined TMS and EEG study . Neuroscience , 331 , 109 – 119 . 10.1016/j.neuroscience.2016.06.015 27318302

[b25] Formann , A. K. , Waldherr , K. , & Piswanger , K. ( 2011 ). Wiener Matrizen-Test 2 (WMT-2): Ein Rasch-Skalierter Sprachfreier Kurztest zur Erfassung der Intelligenz [Viennese Matrices Test 2 (WMT-2): A rapid-scaled, language-free short-circuit test for the assesment of intelligence] . Hogrefe. https://www.hogrefe.com/at/shop/wiener-matrizen-test-2.html

[b26] Francis , H. , & Stevenson , R. ( 2013 ). Validity and test-retest reliability of a short dietary questionnaire to assess intake of saturated fat and free sugars: A preliminary study . Journal of Human Nutrition and Dietetics , 26 ( 3 ), 234 – 242 . 10.1111/jhn.12008 23190372

[b27] Frank , M. J. , Loughry , B. , & O’Reilly , R. C. ( 2001 ). Interactions between frontal cortex and basal ganglia in working memory: A computational model . Cognitive, Affective, & Behavioral Neuroscience , 1 ( 2 ), 137 – 160 . 10.3758/cabn.1.2.137 12467110

[b29] Fromm , S. P. , & Horstmann , A. ( 2019 ). Psychometric evaluation of the german version of the dietary fat and free sugar-short questionnaire . Obesity Facts , 12 ( 5 ), 518 – 528 . https://doi.org/10.1159/000501969 31553993 10.1159/000501969 PMC6876588

[b30] Fuscà , M. , Siebenhühner , F. , Wang , S. H. , Myrov , V. , Arnulfo , G. , Nobili , L. , & Palva , S. ( 2023 ). Brain criticality predicts individual levels of inter-areal synchronization in human electrophysiological data . Nature Communications , 14 ( 1 ), 4736 . 10.1038/s41467-023-40056-9 PMC10406818 37550300

[b31] Gao , R. , Peterson , E. J. , & Voytek , B. ( 2017 ). Inferring synaptic excitation/inhibition balance from field potentials . NeuroImage , 158 , 70 – 78 . 10.1016/j.neuroimage.2017.06.078 28676297

[b32] Ghani , U. , Signal , N. , Niazi , I. K. , & Taylor , D. ( 2020 ). ERP based measures of cognitive workload: A review . Neuroscience & Biobehavioral Reviews , 118 , 18 – 26 . 10.1016/j.neubiorev.2020.07.020 32707343

[b33] Gray , H. M. , Ambady , N. , Lowenthal , W. T. , & Deldin , P. ( 2004 ). P300 as an index of attention to self-relevant stimuli . Journal of Experimental Social Psychology , 40 ( 2 ), 216 – 224 . 10.1016/s0022-1031(03)00092-1

[b34] Hardstone , R. , Poil , S. S. , Schiavone , G. , Jansen , R. , Nikulin , V. V. , Mansvelder , H. D. , & Linkenkaer-Hansen , K. ( 2012 ). Detrended fluctuation analysis: A scale-free view on neuronal oscillations . Frontiers in Physiology , 3 , 23105 . 10.3389/fphys.2012.00450 PMC3510427 23226132

[b35] Hartmann , H. , Janssen , L. K. , Herzog , N. , Morys , F. , Fängström , D. , Fallon , S. J. , & Horstmann , A. ( 2023 ). Self-reported intake of high-fat and high-sugar diet is not associated with cognitive stability and flexibility in healthy men . Appetite , 183 , 106477 . 10.1016/j.appet.2023.106477 36764221

[b36] Hartmann , H. , Pauli , L. K. , Janssen , L. K. , Huhn , S. , Ceglarek , U. , & Horstmann , A. ( 2020 ). Preliminary evidence for an association between intake of high‐fat high‐sugar diet, variations in peripheral dopamine precursor availability and dopamine‐dependent cognition in humans . Journal of Neuroendocrinology , 32 ( 12 ), e12917 . 10.1111/jne.12917 33270945

[b37] Hazy , T. E. , Frank , M. J. , & O’Reilly , R. C. ( 2006 ). Banishing the homunculus: Making working memory work . Neuroscience , 139 ( 1 ), 105 – 118 . 10.1016/j.neuroscience.2005.04.067 16343792

[b38] Herzog , N. , Hartmann , H. , Janssen , L. K. , Waltmann , M. , Fallon , S. J. , Deserno , L. , & Horstmann , A. ( 2024a ). Working memory gating in obesity: Insights from a case-control fMRI study . Appetite , 195 , 107179 . 10.1016/j.appet.2023.107179 38145879

[b39] Herzog , N. , Hartmann , H. , Janssen , L. K. , Kanyamibwa , A. , Waltmann , M. , Kovacs , P. , Deserno , L. , Fallon , S. J. , Villringer , A. , & Horstmann , A. ( 2024b ). Working memory gating in obesity is moderated by striatal dopaminergic gene variants . eLife , 13 , RP93369 . 10.7554/elife.93369.3 39431987 PMC11493406

[b40] Herzog , N. D. , Steinfath , T. P. , & Tarrasch , R. ( 2021 ). Critical dynamics in spontaneous resting-state oscillations are associated with the attention-related P300 ERP in a Go/Nogo task . Frontiers in Neuroscience , 15 , 632922 . 10.3389/fnins.2021.632922 33828446 PMC8019703

[b41] Huang , Y. , Parra , L. C. , & Haufe , S. ( 2016 ). The New York Head—A precise standardized volume conductor model for EEG source localization and tES targeting . NeuroImage , 140 , 150 – 162 . 10.1016/j.neuroimage.2015.12.019 26706450 PMC5778879

[b42] Irrmischer , M. , Poil , S. S. , Mansvelder , H. D. , Intra , F. S. , & Linkenkaer‐Hansen , K. ( 2017 ). Strong long‐range temporal correlations of beta/gamma oscillations are associated with poor sustained visual attention performance . European Journal of Neuroscience , 48 ( 8 ), 2674 – 2683 . 10.1111/ejn.13672 28858404 PMC6221163

[b43] Jäger , A. T. P. , Bailey , A. , Huntenburg , J. M. , Tardif , C. L. , Villringer , A. , Gauthier , C. J. , Nikulin , V. , Bazin , P.-L. , & Steele , C. J. ( 2024 ). Decreased long‐range temporal correlations in the resting‐state functional magnetic resonance imaging blood‐oxygen‐level‐dependent signal reflect motor sequence learning up to 2 weeks following training . Human Brain Mapping , 45 ( 4 ), e26539 . 10.1002/hbm.26539 38124341 PMC10915743

[b45] Kapralov , N. , Jamshidi Idaji , M., Stephani , T. , Studenova , A. , Vidaurre , C. , Ros , T. , & Nikulin , V. ( 2023 ). Sensorimotor brain-computer interface performance depends on signal-to-noise ratio but not connectivity of the mu rhythm in a multiverse analysis of longitudinal data . BioRxiv , 2023-09. 10.1101/2023.09.30.558407 39265614

[b202] Kok , A. ( 2001 ). On the utility of P3 amplitude as a measure of processing capacity . Psychophysiology , 38 ( 3 ), 557 – 577 . https://doi.org/10.1017/S0048577201990559 11352145 10.1017/s0048577201990559

[b46] Landau , S. M. , Lal , R. , O’Neil , J. P. , Baker , S. , & Jagust , W. J. ( 2009 ). Striatal dopamine and working memory . Cerebral Cortex , 19 ( 2 ), 445 – 454 . 10.1093/cercor/bhn095 18550595 PMC2733326

[b47] Lendner , J. D. , Helfrich , R. F. , Mander , B. A. , Romundstad , L. , Lin , J. J. , Walker , M. P. , Larsson , P. G. , & Knight , R. T. ( 2020 ). An electrophysiological marker of arousal level in humans . elife , 9 , e55092 . 10.7554/elife.55092 32720644 PMC7394547

[b48] Leyton , M. , Dagher , A. , Boileau , I. , Casey , K. , Baker , G. B. , Diksic , M. , Gunn , R. , Young , S. N. , & Benkelfat , C. ( 2004 ). Decreasing amphetamine-induced dopamine release by acute phenylalanine/ tyrosine depletion: A PET/[11C]raclopride study in healthy men . Neuropsychopharmacology , 29 ( 2 ), 427 – 432 . 10.1038/sj.Npp.1300328 14583741

[b203] Leyton , M. , Young , S. N. , Blier , P. , Ellenbogen , M. A. , Palmour , R. M. , Ghadirian , A. M. , & Benkelfat , C. ( 1997 ). The effect of tryptophan depletion on mood in medication-free, former patients with major affective disorder . Neuropsychopharmacology , 16 ( 4 ), 294 – 297 . 10.1016/S0893-133X(96)00262-X 9094147

[b49] Linkenkaer-Hansen , K. , Monto , S. , Rytsälä , H. , Suominen , K. , Isometsä , E. , & Kähkönen , S. ( 2005 ). Breakdown of long-range temporal correlations in theta oscillations in patients with major depressive disorder . Journal of Neuroscience , 25 ( 44 ), 10131 – 10137 . 10.1523/jneurosci.3244-05.2005 16267220 PMC6725784

[b50] Linkenkaer-Hansen , K. , Nikouline , V. V. , Palva , J. M. , & Ilmoniemi , R. J. ( 2001 ). Long-range temporal correlations and scaling behavior in human brain oscillations . Journal of Neuroscience , 21 ( 4 ), 1370 – 1377 . 10.1523/jneurosci.21-04-01370.2001 11160408 PMC6762238

[b51] Linkenkaer-Hansen , K. , Smit , D. J. , Barkil , A. , van Beijsterveldt , T. E. , Brussaard , A. B. , Boomsma , D. I. , & de Geus , E. J . ( 2007 ). Genetic contributions to long-range temporal correlations in ongoing oscillations . Journal of Neuroscience , 27 ( 50 ), 13882 – 13889 . 10.1523/jneurosci.3083-07.2007 18077700 PMC6673639

[b52] Mahjoory , K. , Cesnaite , E. , Hohlefeld , F. U. , Villringer , A. , & Nikulin , V. V. ( 2019 ). Power and temporal dynamics of alpha oscillations at rest differentiate cognitive performance involving sustained and phasic cognitive control . NeuroImage , 188 , 135 – 144 . 10.1016/j.neuroimage.2018.12.001 30517844

[b53] Maris , E. , & Oostenveld , R. ( 2007 ). Nonparametric statistical testing of EEG- and MEG-data . Journal of Neuroscience Methods , 164 , 177 – 190 . 10.1016/j.jneumeth.2007.03.024 17517438

[b54] Medel , V. , Irani , M. , Crossley , N. , Ossandón , T. , & Boncompte , G. ( 2023 ). Complexity and 1/f slope jointly reflect brain states . Scientific Reports , 13 ( 1 ), 21700 . 10.1038/s41598-023-47316-0 38065976 PMC10709649

[b55] Meisel , C. , Klaus , A. , Vyazovskiy , V. V. , & Plenz , D. ( 2017 ). The interplay between long-and short-range temporal correlations shapes cortex dynamics across vigilance states . Journal of Neuroscience , 37 ( 42 ), 10114 – 10124 . 10.1523/jneurosci.0448-17.2017 28947577 PMC5647769

[b56] Moja , E. A. , Lucini , V. , Benedetti , F. , & Lucca , A. ( 1996 ). Decrease in plasma phenylalanine and tyrosine after phenylalanine-tyrosine free amino acid solutions in man . Life Sciences , 58 ( 26 ), 2389 – 2395 . 10.1016/0024-3205(96)00242-1 8691983

[b58] Montgomery , A. J. , McTavish , S. F. B. , Cowen , P. J. , & Grasby , P. M. ( 2003 ). Reduction of brain dopamine concentration with dietary tyrosine plus phenylalanine depletion: An [11 C]raclopride PET study . American Journal of Psychiatry , 160 ( 10 ), 1887 – 1889 . 10.1176/appi.ajp.160.10.1887 14514507

[b59] Monto , S. , Vanhatalo , S. , Holmes , M. D. , & Palva , J. M. ( 2007 ). Epileptogenic neocortical networks are revealed by abnormal temporal dynamics in seizure-free subdural EEG . Cerebral Cortex , 17 ( 6 ), 1386 – 1393 . 10.1093/cercor/bhl049 16908492

[b60] Moran , J. K. , Michail , G. , Heinz , A. , Keil , J. , & Senkowski , D. ( 2019 ). Long-range temporal correlations in resting state beta oscillations are reduced in schizophrenia . Frontiers in Psychiatry , 10 , 517 . 10.3389/fpsyt.2019.00517 31379629 PMC6659128

[b61] Nakao , T. , Miyagi , M. , Hiramoto , R. , Wolff , A. , Gomez-Pilar , J. , Miyatani , M. , & Northoff , G. ( 2019 ). From neuronal to psychological noise–Long-range temporal correlations in EEG intrinsic activity reduce noise in internally-guided decision making . NeuroImage , 201 , 116015 . 10.1016/j.neuroimage.2019.116015 31306772

[b63] Nikulin , V. V. , & Brismar , T. ( 2004 ). Long-range temporal correlations in alpha and beta oscillations: Effect of arousal level and test–retest reliability . Clinical Neurophysiology , 115 ( 8 ), 1896 – 1908 . 10.1016/j.clinph.2004.03.019 15261868

[b64] Nikulin , V. V. , Jönsson , E. G. , & Brismar , T. ( 2012 ). Attenuation of long-range temporal correlations in the amplitude dynamics of alpha and beta neuronal oscillations in patients with schizophrenia . NeuroImage , 61 ( 1 ), 162 – 169 . 10.1016/j.neuroimage.2012.03.008 22430497

[b65] Nir-Cohen , G. , Kessler , Y. , & Egner , T. ( 2020 ). Neural substrates of working memory updating . Journal of Cognitive Neuroscience , 32 ( 12 ), 2285 – 2302 . 10.1162/jocn_a_01625 32897122

[b66] O’Reilly , R. C. , & Frank , M. J. ( 2006 ). Making working memory work: A computational model of learning in the prefrontal cortex and basal ganglia . Neural Computation , 18 ( 2 ), 283 – 328 . 10.1162/089976606775093909 16378516

[b67] Pathania , A. , Schreiber , M. , Miller , M. W. , Euler , M. J. , & Lohse , K. R. ( 2021 ). Exploring the reliability and sensitivity of the EEG power spectrum as a biomarker . International Journal of Psychophysiology , 160 , 18 – 27 . 10.1016/j.ijpsycho.2020.12.002 33340559

[b68] Pfeffer , T. , Avramiea , A. E. , Nolte , G. , Engel , A. K. , Linkenkaer-Hansen , K. , & Donner , T. H. ( 2018 ). Catecholamines alter the intrinsic variability of cortical population activity and perception . PLoS Biology , 16 ( 2 ), e2003453 . 10.1371/journal.pbio.2003453 29420565 PMC5821404

[b69] Poil , S. S. , Hardstone , R. , Mansvelder , H. D. , & Linkenkaer-Hansen , K. ( 2012 ). Critical-state dynamics of avalanches and oscillations jointly emerge from balanced excitation/inhibition in neuronal networks . Journal of Neuroscience , 32 ( 29 ), 9817 – 9823 . 10.1523/jneurosci.5990-11.2012 22815496 PMC3553543

[b70] Polich , J. ( 2007 ). Updating P300: An integrative theory of P3a and P3b . Clinical Neurophysiology , 118 ( 10 ), 2128 – 2148 . 10.1016/j.clinph.2007.04.019 17573239 PMC2715154

[b71] Ranganath , A. , & Jacob , S. N. ( 2016 ). Doping the mind: Dopaminergic modulation of prefrontal cortical cognition . The Neuroscientist , 22 ( 6 ), 593 – 603 . 10.1177/1073858415602850 26338491

[b204] Rcore Team . ( 2015 ). R: a language and environment for statistical computing, 2021.

[b205] RStudio Team . ( 2016 ). RStudio: Integrated Development Environment for R. RStudio, Inc. https://www.rstudio.com/

[b72] Samek , W. , Blythe , D. A. , Curio , G. , Müller , K. R. , Blankertz , B. , & Nikulin , V. V. ( 2016 ). Multiscale temporal neural dynamics predict performance in a complex sensorimotor task . NeuroImage , 141 , 291 – 303 . 10.1016/j.neuroimage.2016.06.056 27402598

[b73] Scolari , M. , Seidl-Rathkopf , K. N. , & Kastner , S. ( 2015 ). Functions of the human frontoparietal attention network: Evidence from neuroimaging . Current Opinion in Behavioral Sciences , 1 , 32 – 39 . 10.1016/j.cobeha.2014.08.003 27398396 PMC4936532

[b74] Shew , W. L. , Yang , H. , Yu , S. , Roy , R. , & Plenz , D. ( 2011 ). Information capacity and transmission are maximized in balanced cortical networks with neuronal avalanches . Journal of Neuroscience , 31 ( 1 ), 55 – 63 . 10.1523/jneurosci.4637-10.2011 21209189 PMC3082868

[b75] Shew , W. L. , & Plenz , D. ( 2013 ). The functional benefits of criticality in the cortex . The Neuroscientist , 19 ( 1 ), 88 – 100 . 10.1177/1073858412445487 22627091

[b76] Simola , J. , Zhigalov , A. , Morales-Muñoz , I. , Palva , J. M. , & Palva , S. ( 2017 ). Critical dynamics of endogenous fluctuations predict cognitive flexibility in the Go/NoGo task . Scientific Reports , 7 ( 1 ), 2909 . 10.1038/s41598-017-02750-9 28588303 PMC5460255

[b207] Smit , D. J. A. , Linkenkaer-Hansen , K. , & de Geus , E. J. C . ( 2013 ). Long-range temporal correlations in resting-state α oscillations predict human timing-error dynamics . The Journal of Neuroscience , 33 ( 27 ), 11212 – 11220 . 10.1523/JNEUROSCI.2816-12.2013 23825424 PMC6618606

[b208] Stam , C. J. , & De Bruin , E. A. ( 2004 ). Scale‐free dynamics of global functional connectivity in the human brain . Human Brain Mapping , 22 ( 2 ), 97 – 109 . 10.1002/hbm.20016 15108297 PMC6871799

[b77] Strobel , A. , Beauducel , A. , Debener , S. , & Brocke , B. ( 2006 ). Eine deutschsprachige Version des BIS/BAS-Fragebogens von Carver und White . 10.1024//0170-1789.22.3.216

[b78] Tanaka , S. ( 2006 ). Dopaminergic control of working memory and its relevance to schizophrenia: A circuit dynamics perspective . Neuroscience , 139 ( 1 ), 153 – 171 . 10.1016/j.neuroscience.2005.08.070 16324800

[b79] Thompson , C. G. , Kim , R. S. , Aloe , A. M. , & Becker , B. J. ( 2017 ). Extracting the variance inflation factor and other multicollinearity diagnostics from typical regression results . Basic and Applied Social Psychology , 39 ( 2 ), 81 – 90 . 10.1080/01973533.2016.1277529

[b80] Uitvlugt , M. G. , Pleskac , T. J. , & Ravizza , S. M. ( 2016 ). The nature of working memory gating in Parkinson’s disease: A multi-domain signal detection examination . Cognitive, Affective, & Behavioral Neuroscience , 16 ( 2 ), 289 – 301 . 10.3758/s13415-015-0389-9 26518210

[b81] van Schouwenburg , M. , Aarts , E. , & Cools , R. ( 2010 ). Dopaminergic modulation of cognitive control: Distinct roles for the prefrontal cortex and the basal ganglia . Current Pharmaceutical Design , 16 ( 18 ), 2026 – 2032 . 10.2174/138161210791293097 20370667

[b206] Verleger , R. ( 1988 ). Event-related potentials and cognition: A critique of the context updating hypothesis and an alternative interpretation of P3 . Behavioral and Brain Sciences , 11 ( 3 ), 343 – 356 . 10.1017/S0140525X00058015

[b82] Vogt , B. A. ( 2019 ). Cingulate cortex in the three limbic subsystems . Handbook of Clinical Neurology , 166 , 39 – 51 . 10.1016/b978-0-444-64196-0.00003-0 31731924

[b84] Wechsler , D. ( 2008 ). Wechsler adult intelligence scale–Fourth Edition (WAIS–IV) . NCS Pearson , 22 ( 498 ), 1 . 10.53841/bpstest.2010.wais4

[b85] Wittchen , H. U. , Zaudig , M. , & Fydrich , T. ( 1997 ). SKID. Strukturiertes klinisches Interview für DSM-IV . Achse I und II. Handanweisung. 10.1026//0084-5345.28.1.68

[b86] Zanto , T. P. , & Gazzaley , A. ( 2013 ). Fronto-parietal network: Flexible hub of cognitive control . Trends in Cognitive Sciences , 17 ( 12 ), 602 – 603 . 10.1016/j.tics.2013.10.001 24129332 PMC3873155

[b87] Zsido , R. G. , Molloy , E. N. , Cesnaite , E. , Zheleva , G. , Beinhölzl , N. , Scharrer , U. , Piecha , F. A. , Regenthal , R. , Villringer , A. , Nikulin , V. V. , & Sacher , J. ( 2022 ). One‐week escitalopram intake alters the excitation–inhibition balance in the healthy female brain . Human Brain Mapping , 43 ( 6 ), 1868 – 1881 . 10.1002/hbm.25760 35064716 PMC8933318

