# Supplementary material for: Balancing excitation and inhibition: The role of neural network dynamics in working memory gating
Source: Imaging Neurosci (Camb). 2024 Dec 2;2:imag-2-00380. doi: 10.1162/imag_a_00380 (PMC12315747; doi:10.1162/imag_a_00380)
Supplement: Supplementary Material [file imag_a_00380-supp.pdf]

SUPPLEMENTS

Transparency statement

We deviate from our initial pre-registered analyses in several ways. First, we refrained from further exploratory analysis of Single Nucleotide Polymorphism (SPN) effects, as initially put forward in our pre-registration. Recent studies conducted in our lab, published after the pre-registration, indicate that SNPs impact WM performance on our task in combination with high BMI only. Since our sample consists of individuals with normal weight, we decided to limit our analyses to blood amino acid ratios, as previous research has shown these ratios to influence task performance in a sample with a similar BMI range (Herzog et al., 2024). Secondly, we opted to use the PSD slope instead of fE/I (measure developed by Bruining et al., 2020) as a measure of the E/I ratio. We did so because PSD slope is a more established measure, hence providing a more reliable assessment.

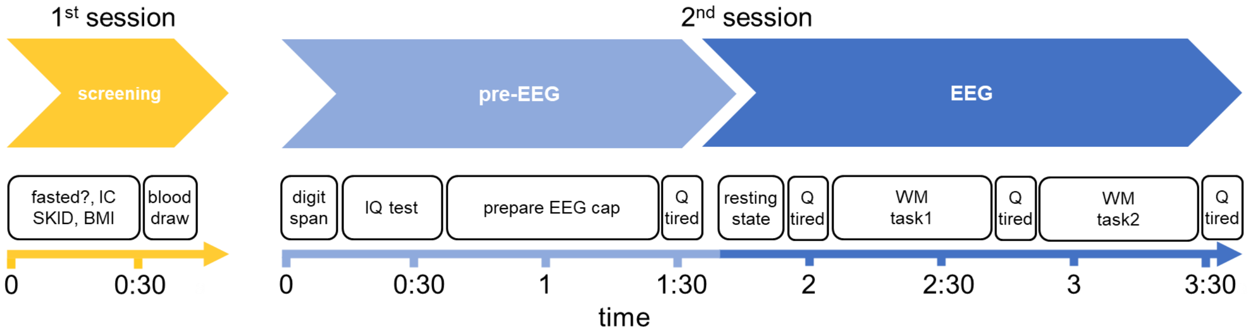

Figure S1. Schematic illustration of the study design. On the first day, participants underwent the screening session where in- and exclusion criteria were checked, i.e. BMI and depression (using the SKID interview). After inclusion, serum blood samples were taken in order to extract information on amino acid profiles indicative of central blood amino acid ratio. Participants, therefore, came overnight-fasted. On the second test day, participants first did two neuropsychological tests: the digit span task, and an IQ Test. After that, participants were prepared for the EEG. Before they did the resting-state EEG, participants were first inclined about their state of tiredness and well-being. Then they proceeded to do a 10-minute eyes-closed rest EEG recording and then inclined about their state of tiredness and well-being again. Participants then did the two working memory tasks, between which they were asked about their state of tiredness and well-being again.

Table S1. Points that needed to be mentioned after the training.

● remember target items (marked by T)
● ignore NON-target items (marked by N)
● remember ONLY LAST 2 seen targets (in case there are two Ts after each other)

Table S2. *Effect sizes and p-values for the two-way interaction of model 1 and 2, and the three-way interaction of model 1a and* 2a

	DFA*condition		DFA*condition*AA		PSD*condition		PSD*condition*AA	
	p-value	X^2	p-value	X^2	p-value	X^2	p-value	X^2
Fp1	0.121	5.811	0.590	1.917	0.702	1.416	0.106	6.108
Fp2	0.127	5.696	0.576	1.982	0.147	5.371	0.285	3.794
F7	0.051	7.784	0.604	1.850	0.468	2.542	**0.014**	**10.605**
F3	0.101	6.220	0.423	2.802	0.639	1.690	0.056	7.544
Fz	0.080	6.770	0.425	2.792	0.197	4.675	0.068	7.140
F4	0.057	7.531	0.435	2.733	0.800	1.006	**0.011**	**11.093**
F8	0.120	5.828	0.344	3.324	0.414	2.860	0.131	5.622
FC5	0.154	5.254	0.423	2.800	0.952	0.342	**0.045**	**8.038**
FC1	**0.049**	**7.821**	0.330	3.427	0.375	3.108	**0.015**	**10.430**
FC2	0.135	5.556	0.280	3.830	0.198	4.661	**0.018**	**10.029**
FC6	0.116	5.904	0.219	4.430	0.939	0.406	0.087	6.569
T7	0.131	5.624	0.403	2.929	0.801	1.002	0.162	5.141
C3	0.300	3.663	0.663	1.585	0.583	1.949	0.065	7.240
Cz	0.330	3.433	0.416	2.846	**0.049**	7.844	0.068	7.135
C4	**0.018**	**10.029**	0.736	1.271	0.607	1.836	**0.004**	**13.084**
T8	0.345	3.322	0.292	3.729	0.366	3.172	0.300	3.662
CP5	0.738	1.260	0.717	1.353	0.609	1.826	0.162	5.137
CP1	0.094	6.399	0.751	1.208	0.285	3.787	0.099	6.282
CP2	0.053	7.666	0.444	2.678	0.100	6.261	0.108	6.076
CP6	**0.049**	**7.818**	0.140	5.484	0.213	4.494	0.050	7.792
AFz	0.061	7.356	0.462	2.573	0.501	2.362	0.175	4.955
P7	0.235	4.258	0.265	3.965	0.155	5.233	0.159	5.174
P3	0.229	4.320	0.338	3.371	0.269	3.931	0.085	6.613
Pz	0.176	4.944	0.749	1.218	0.116	5.902	0.208	4.546
P4	0.062	7.340	0.243	4.172	0.173	4.986	**0.037**	**8.462**
P8	**0.042**	**8.225**	0.083	6.681	0.180	4.891	**0.029**	**9.037**
PO9	0.369	3.151	0.119	5.846	0.261	4.006	0.150	5.320
O1	0.219	4.429	0.132	5.620	0.271	3.917	0.065	7.237
Oz	0.255	4.057	0.335	3.395	0.486	2.442	0.092	6.441
O2	0.103	6.193	0.334	3.398	0.541	2.153	**0.028**	**9.061**
PO10	**0.039**	**8.347**	0.278	3.854	**0.028**	9.102	**0.010**	**11.340**
AF7	0.064	7.264	0.578	1.975	0.115	5.939	0.065	7.231
AF3	0.068	7.134	0.491	2.414	0.461	2.581	0.085	6.623
AF4	0.100	6.243	0.485	2.448	0.173	4.985	0.266	3.958
AF8	0.124	5.766	0.419	2.825	0.512	2.302	0.293	3.720
F5	0.103	6.194	0.490	2.418	0.719	1.341	0.127	5.709
F1	0.066	7.195	0.410	2.880	0.506	2.333	0.053	7.673
F2	0.105	6.146	0.376	3.103	0.508	2.323	0.075	6.902
F6	0.084	6.658	0.412	2.869	0.760	1.172	0.150	5.320
FT7	0.063	7.303	0.472	2.518	0.474	2.508	**0.037**	**8.483**
FC3	0.071	7.028	0.330	3.429	0.773	1.114	**0.020**	**9.890**
FC4	**0.036**	**8.516**	0.259	4.019	0.826	0.899	**0.008**	**11.865**
FT8	0.243	4.177	0.181	4.872	0.334	3.400	**0.042**	**8.184**
C5	0.358	3.224	0.588	1.924	0.983	0.163	0.222	4.395
C1	**0.036**	**8.550**	0.328	3.442	0.218	4.435	**0.020**	**9.881**
C2	**0.038**	**8.434**	0.409	2.886	0.100	6.251	**0.032**	**8.803**
C6	0.127	5.706	0.267	3.951	0.516	2.281	**0.049**	**7.837**
TP7	0.143	5.423	0.325	3.465	0.783	1.074	0.128	5.677
CP3	0.612	1.812	0.856	0.774	0.621	1.771	0.073	6.967
CPz	0.214	4.483	0.648	1.652	0.150	5.320	0.170	5.023
CP4	**0.035**	**8.597**	0.369	3.150	0.230	4.307	**0.024**	**9.425**
TP8	0.189	4.781	0.107	6.100	0.188	4.790	**0.042**	**8.215**
P5	0.215	4.474	0.297	3.688	0.176	4.947	0.098	6.292
P1	0.102	6.207	0.522	2.250	0.123	5.769	0.201	4.624
P2	0.095	6.378	0.441	2.695	0.117	5.898	0.112	5.985
P6	**0.020**	**9.841**	0.168	5.058	0.121	5.806	**0.013**	**10.748**
PO7	0.137	5.520	0.148	5.355	0.158	5.188	0.105	6.135
PO3	0.117	5.887	0.185	4.828	0.081	6.743	0.058	7.501
POz	0.296	3.698	0.675	1.531	0.178	4.920	0.107	6.098
PO4	0.139	5.501	0.584	1.943	0.210	4.526	**0.027**	**9.142**
PO8	0.054	7.636	0.204	4.596	0.421	2.815	**0.032**	**8.797**

Note: Bold values highlight $p<0.05$, uncorrected; DFA = Detrended Fluctuation Analysis (exponent); PSD = Power Spectral Density (slope); AA = Amino Acid (ratio)

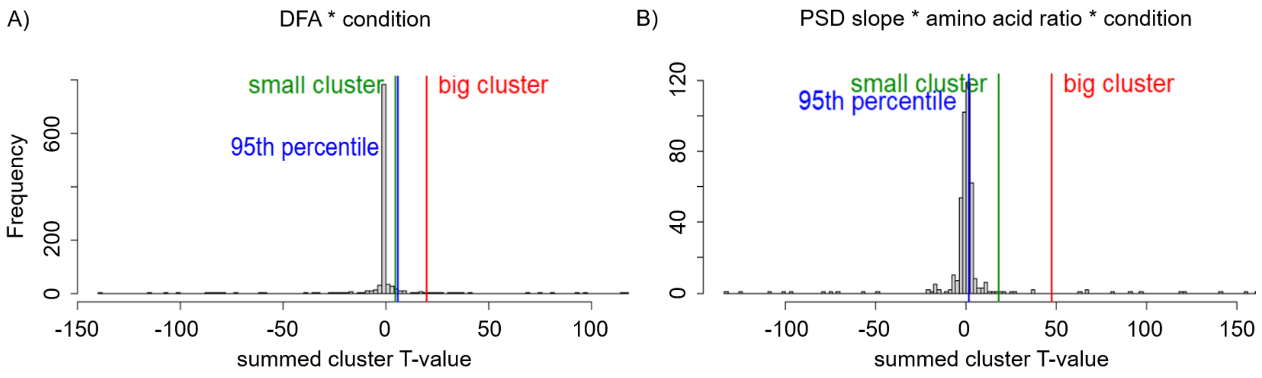

Figure S2. Results of Cluster-Based Permutation Testing. A) DFA-condition interaction: The large cluster (red) exceeds the 95th percentile of the cluster summed T-values distribution, indicating statistical significance ($p < 0.05$). The small cluster (green) falls within the 95th percentile, indicating it is not statistically significant ($p > 0.05$). **B)** PSD slope – amino acid ratio – condition interaction: Both the large (red) and small (green) clusters exceed the 95th percentile of the cluster summed T-values distribution, indicating statistical significance ($p < 0.05$).

Table S3. Output for model with average cluster DFA and control variables.

term	X^2	df	p.value
(Intercept)	269.390	1	0.000
condition	7.092	3	0.069
**DFA**	**4.416**	**1**	**0.036**
**gender**	**5.068**	**1**	**0.024**
zWM_tired	2.933	1	0.087
zWM_conc	0.009	1	0.923
zIQ	3.636	1	0.057
zAge	0.715	1	0.398
zDFS	0.170	1	0.680
**zRelAlphaPow_rest**	**4.840**	**1**	**0.028**
zBIS	1.971	1	0.160
zBAS	0.070	1	0.791
zBMI	1.341	1	0.247
**zDSBack**	**5.138**	**1**	**0.023**
**condition:DFA**	**9.914**	**3**	**0.019**

Table S4. Significant vertices ($p < 0.01$, uncorrected) from the source-level analyses

DFA*condition		PSD*amino acid ratio*condition	
N vertex	area	N vertex	area
15	Right Superior Frontal Gyrus	20	Left Precentral Gyrus
11	Right Middle Frontal Gyrus	17	Left Superior Frontal Gyrus
8	Right Supramarginal Gyrus, posterior	12	Right Precentral Gyrus
6	Right Precentral Gyrus	12	Right Postcentral Gyrus
5	Right Supplementary Motor Cortex	7	Right Middle Frontal Gyrus
5	Right Planum Temporale	6	Left Postcentral Gyrus
4	Left Insular Cortex	6	Right Supramarginal Gyrus, anterior
4	Right Postcentral Gyrus	5	Left Supplementary Motor Cortex
3	Left Superior Frontal Gyrus	4	Right Inferior Frontal Gyrus
3	Right Paracingulate Gyrus	4	Right Supramarginal Gyrus, posterior
3	Right Parietal Operculum Cortex	4	Right Frontal Operculum Cortex
2	Right Frontal Pole	3	Left Middle Frontal Gyrus
2	Right Supramarginal Gyrus, anterior	3	Right Precuneous Cortex
1	Left Frontal Orbital Cortex	3	Right Parietal Operculum Cortex
1	Left Frontal Operculum Cortex	2	Left Subcallosal Cortex
1	Right Superior Temporal Gyrus, posterior	2	Left Precuneous Cortex
		2	Right Inferior Frontal Gyru
		2	Right Angular Gyrus
		2	Right Subcallosal Cortex
		2	Right Cingulate Gyrus, posterior
		2	Right Frontal Orbital Cortex
		1	Left Cingulate Gyrus, anterior
		1	Left Cingulate Gyrus, posterior
		1	Left Frontal Orbital Cortex
		1	Left Parahippocampal Gyrus, anterior
		1	Left Parietal Operculum Cortex
		1	Right Frontal Pole
		1	Right Superior Frontal Gyrus
		1	Right Inferior Temporal Gyrus, temporooccipital
		1	Right Lateral Occipital Cortex, inferior
		1	Right Intracalcarine Cortex
		1	Right Supplementary Motor Cortex
		1	Right Cingulate Gyrus, anterior
		1	Right Parahippocampal Gyrus, anterior
		1	Right Parahippocampal Gyrus, posterior
		1	Right Lingual Gyrus
		1	Right Temporal Fusiform Cortex, posterior
		1	Right Temporal Occipital Fusiform Cortex
		1	Right Central Opercular Cortex
		1	Right Heschl's Gyrus (includes H1 and H2)

Table S5. Output for model with average cluster PSD slope and control variables.

term	X²	df	p.value
(Intercept)	0.535	1	0.464
**condition**	11.496	3	**0.009**
**PSD**	4.352	1	**0.037**
**AAratio**	5.484	1	**0.019**
**gender**	3.800	1	**0.051**
zWM_tired	2.775	1	0.096
zWM_conc	0.000	1	0.984
zIQ	2.911	1	0.088
zAge	0.750	1	0.386
zDFS	0.097	1	0.755
**zRelAlphaPow_rest**	**5.299**	**1**	**0.021**
zBIS	1.702	1	0.192
zBAS	0.065	1	0.798
zBMI	0.476	1	0.490
**zDSBack**	**4.286**	**1**	**0.038**
**condition:PSD**	10.432	3	0.015
**AAratio:PSD**	12.111	3	0.007
**condition:AAratio**	5.167	1	0.023
**condition:PSD:AAratio**	11.414	3	0.010

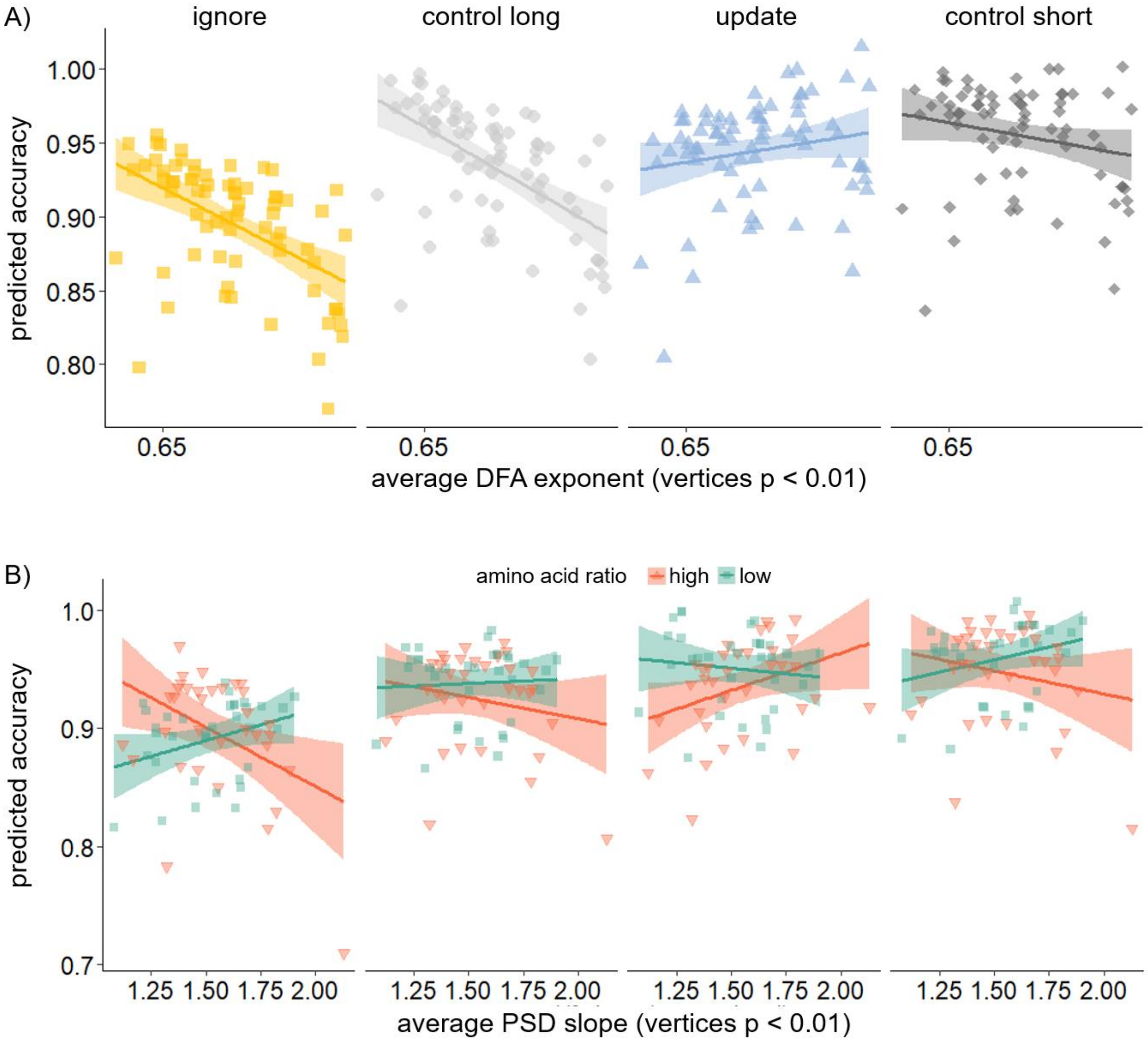

Figure S3. Results for post-hoc test for source-level analysis of A) DFA and B) PSD slope – amino acid ratio effects. Both interactions show a similar condition-specific effect, as on the sensor level.

Table S6. Output for model investigating the interaction of PSD slope and DFA exponent

term	X²	df	p-value
(Intercept)	0.416	1	0.519
**DFA**	**4.610**	**1**	**0.032**
**PSD**	**5.352**	**1**	**0.021**
condition	0.318	1	0.573
**DFA:PSD**	**5.741**	**1**	**0.017**
DFA:condition	0.361	1	0.548
PSD:condition	0.520	1	0.471
DFA:PSD:condition	0.690	1	0.406

Table S7. Output for model investigating the difference in P300 amplitude per condition.

term	estimate	SE	df	t value	p-value
(Intercept)	1.6574	0.1554	72.7468	10.667	< 0.000
condition	-0.4485	0.1207	71.5123	-3.716	0.000398***

Table S8. Output for model investigating P300 effects on condition-dependent performance.

term	X²	df	p.value
(Intercept)	6.480	1	0.011
**condition**	**28.292**	**1**	**<0.000**
P300	0.004	1	0.947
AAratio	0.324	1	0.569
zWM_tired	2.484	1	0.115
zWM_conc	0.058	1	0.810
zIQ	1.697	1	0.193
zAge	0.068	1	0.794
zDFS	0.021	1	0.884
zRelAlphaPow_rest	0.979	1	0.323
zBIS	0.001	1	0.977
zBAS	0.413	1	0.520
zBMI	0.031	1	0.861
zDSBack	1.027	1	0.311
**condition:P300**	**4.243**	**1**	**0.039**

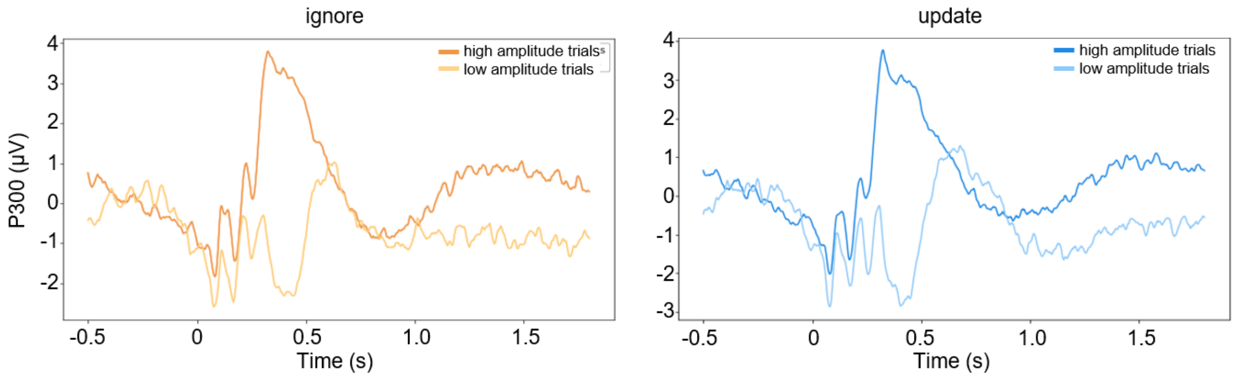

Figure S4. High vs. low amplitude trials for ignore **(A)** and update **(B)** condition. The average amplitude in high amplitude trials (darker lines) has the typical amplitude peak at ~ 300 msec, where low amplitude trials (brighter lines) display a shift in latency, peaking after ~ 500 msec only.

Table S9. Output for model investigating the effect of DFA on condition-dependent P300 amplitude

term	X^2	df	p.value
(Intercept)	6.534	1	0.011
DFA	1.193	1	0.275
condition	4.145	1	0.042
DFA:condition	2.118	1	0.146

Table S10. Output for model investigating the effect of PSD slope and amino acid ratio on condition-dependent P300 amplitude

term	X^2	df	p.value
(Intercept)	0.162	1	0.687
PSD	0.179	1	0.672
AAratio	0.149	1	0.700
condition	1.475	1	0.225
PSD:AAratio	0.062	1	0.804
PSD:condition	0.855	1	0.355
AAratio:condition	1.658	1	0.198
PSD:AAratio:condition	1.124	1	0.289

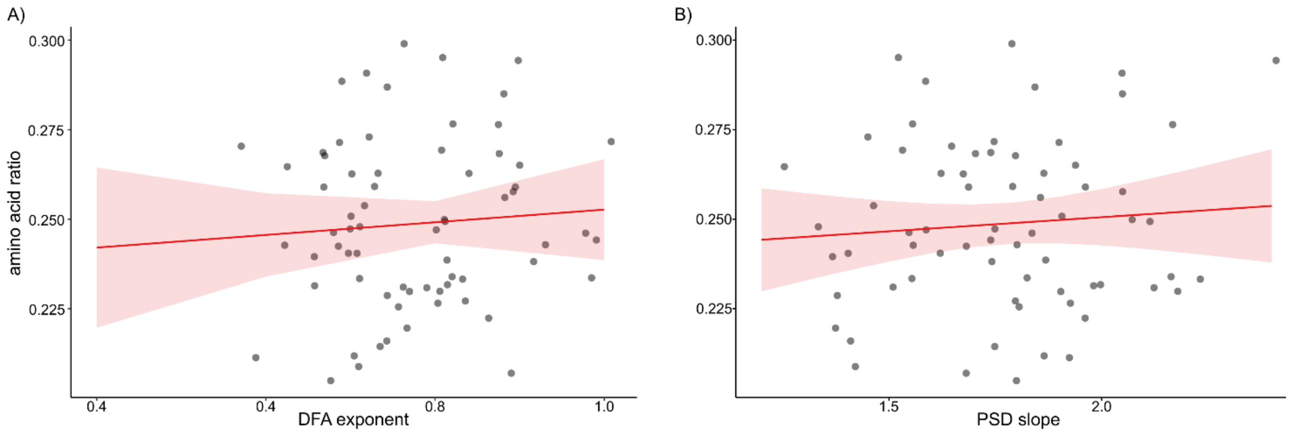

Figure S5. Direct relationship of amino acid ratio with resting-state E/I measures. Neither DFA exponent (A) and nor PSD slope (B) correlate significantly with blood amino acid ratio.
